# Human-Brain-Derived Ischemia-Induced Stem Cell Transplantation Is Associated with a Greater Neurological Functional Improvement Compared with Human-Bone Marrow-Derived Mesenchymal Stem Cell Transplantation in Mice After Stroke

**DOI:** 10.3390/ijms252212065

**Published:** 2024-11-10

**Authors:** Shuichi Tanada, Takayuki Nakagomi, Akiko Nakano-Doi, Toshinori Sawano, Shuji Kubo, Yoji Kuramoto, Kazutaka Uchida, Kenichi Yamahara, Nobutaka Doe, Shinichi Yoshimura

**Affiliations:** 1 Department of Neurosurgery, Hyogo Medical University, 1-1 Mukogawacho, Nishinomiya 663-8501, Japan; ds2114@hyo-med.ac.jp (S.T.); yo-kuramoto@hyo-med.ac.jp (Y.K.); kuchida@hyo-med.ac.jp (K.U.); hyogoneuro@yahoo.co.jp (S.Y.); 2 Institute for Advanced Medical Sciences, Hyogo Medical University (Nishinomiya Campus), 1-1 Mukogawacho, Nishinomiya 663-8501, Japan; nakano@hyo-med.ac.jp (A.N.-D.); s-kubo@hyo-med.ac.jp (S.K.); yamahara@hyo-med.ac.jp (K.Y.); 3 Department of Therapeutic Progress in Brain Diseases, Hyogo Medical University, 1-1 Mukogawacho, Nishinomiya 663-8501, Japan; 4 Department of Biomedical Sciences, Ritsumeikan University, 1-1-1 Nojihigashi, Kusatsu 525-8577, Japan; t-sawano@fc.ritsumei.ac.jp; 5 Department of Rehabilitation, Hyogo Medical University (Kobe Campus), 1-3-6 Minatojima, Chuo-ku, Kobe 650-8530, Japan; doe@hyo-med.ac.jp

**Keywords:** ischemic stroke, cell transplantation, neural stem/progenitor cells, injury/ischemia-induced stem cells, mesenchymal stem cells, neurological function

## Abstract

The transplantation of injury/ischemia-induced stem cells (iSCs) extracted from post-stroke human brains can improve the neurological functions of mice after stroke. However, the usefulness of iSCs as an alternative stem cell source remains unclear. The current study aimed to assess the efficacy of iSC and mesenchymal stem cell (MSC) transplantation. In this experiment, equal numbers of human brain-derived iSCs (h-iSCs) (5.0 × 10^4^ cells/μL) and human bone marrow-derived MSCs (h-MSCs) (5.0 × 10^4^ cells/μL) were intracranially transplanted into post-stroke mouse brains after middle cerebral artery occlusion. Results showed that not only h-iSC transplantation but also h-MSC transplantation activated endogenous neural stem/progenitor cells (NSPCs) around the grafted sites and promoted neurological functional improvement. However, mice that received h-iSC transplantation experienced improvement in a higher number of behavioral tasks compared with those that received h-MSC transplantation. To investigate the underlying mechanism, NSPCs extracted from the ischemic areas of post-stroke mouse brains were cocultured with h-iSCs or h-MSCs. After coincubation, NSPCs, h-iSCs, and h-MSCs were selectively collected via fluorescence-activated cell sorting. Next, their traits were analyzed via microarray analysis. The genes related to various neuronal lineages in NSPCs after coincubation with h-iSCs were enriched compared with those in NSPCs after coincubation with h-MSCs. In addition, the gene expression patterns of h-iSCs relative to those of h-MSCs showed that the expression of genes related to synapse formation and neurotransmitter-producing neurons increased more after coincubation with NSPCs. Hence, cell–cell interactions with NSPCs promoted transdifferentiation toward functional neurons predominantly in h-iSCs. In accordance with these findings, immunohistochemistry showed that the number of neuronal networks between NSPCs and h-iSCs was higher than that between NSPCs and h-MSCs. Therefore, compared with h-MSC transplantation, h-iSC transplantation is associated with a higher neurological functional improvement, presumably by more effectively modulating the fates of endogenous NSPCs and grafted h-iSCs themselves.

## 1. Introduction

Cell transplantation is a promising therapeutic option for central nervous system diseases, such as ischemic stroke. Various cell types, such as induced pluripotent stem (iPS) cells [1], neural stem/progenitor cells (NSPCs) [2], mesenchymal stem cells (MSCs) [3], and bone marrow mononuclear cells [4], are proposed as candidates for cell sources in preclinical studies. However, the cell types that are most effective for treating ischemic stroke remains unclear.

Injury/ischemia-induced stem cells (iSCs) are unique stem cells that are originally detected within the ischemic areas of post-stroke mouse brains [5]. The actual traits of iSCs remain unclear. Nevertheless, mouse-derived iSCs are broadly categorized into the following two subtypes: stem cells with neural stem/progenitor cell activities (injury/ischemia-induced NSPCs) [5,6] and stem cells with multipotency that can differentiate into both neural and non-neural lineages, such as mesenchymal lineages [7]. In addition, based on a previous study, iSCs with multipotency were present in the brain ischemic areas of not only mice [7] but also patients with stroke [8]. Further, human-derived iSCs (h-iSCs) can differentiate into electrophysiologically functional neurons [8]. According to a recent study, h-iSC transplantation into post-stroke mice was associated with an improvement in neurological dysfunction, presumably via various mechanisms, such as the activation of endogenous NSPCs, neuronal replacement, and neural network formation [9].

In relation to h-iSCs, MSC transplantation is a promising therapy for brain injuries, including ischemic stroke [10]. Several studies have revealed that MSC transplantation after ischemic stroke can promote brain repair via various mechanisms. These include neuronal replacement [11], immunomodulatory effects [12,13], vasculogenesis [14], and MSC-derived factors (e.g., neurotrophic factors [15], extracellular vesicle [13,16], and microRNA [17]). According to a recent study, the transplantation of bone-marrow-derived human MSCs (h-MSCs) into post-stroke mice activated locally derived endogenous NSPCs and improved neurological functions [18].

The difference between h-iSCs and h-MSCs remains unclear. However, both h-iSCs and h-MSCs can differentiate into neural and mesenchymal lineages in vitro [8]. However, compared with h-MSCs, h-iSCs have a higher neurogenic potential in vitro [8]. Hence, in addition to h-MSC transplantation, h-iSC transplantation can be an alternative stem cell source. To further elucidate this notion, the current study aimed to assess the efficacy of h-iSC and h-MSC transplantation. In this experiment, similar numbers of h-iSCs and h-MSCs were intracranially transplanted into post-stroke mouse brains after middle cerebral artery occlusion (MCAO). In addition, locally derived endogenous NSPCs extracted from the ischemic areas were cocultured with h-iSCs or h-MSCs. Then, the changes in gene expression patterns in NSPCs, h-iSCs, and h-MSCs were investigated via microarray analysis, and the formation of neural networks was examined via immunohistochemistry.

## 2. Results

### 2.1. Activation of Endogenous Neural Stem Cells Around the Grafted Cells After h-iSC and h-MSC Transplantation

**Figure 1:**
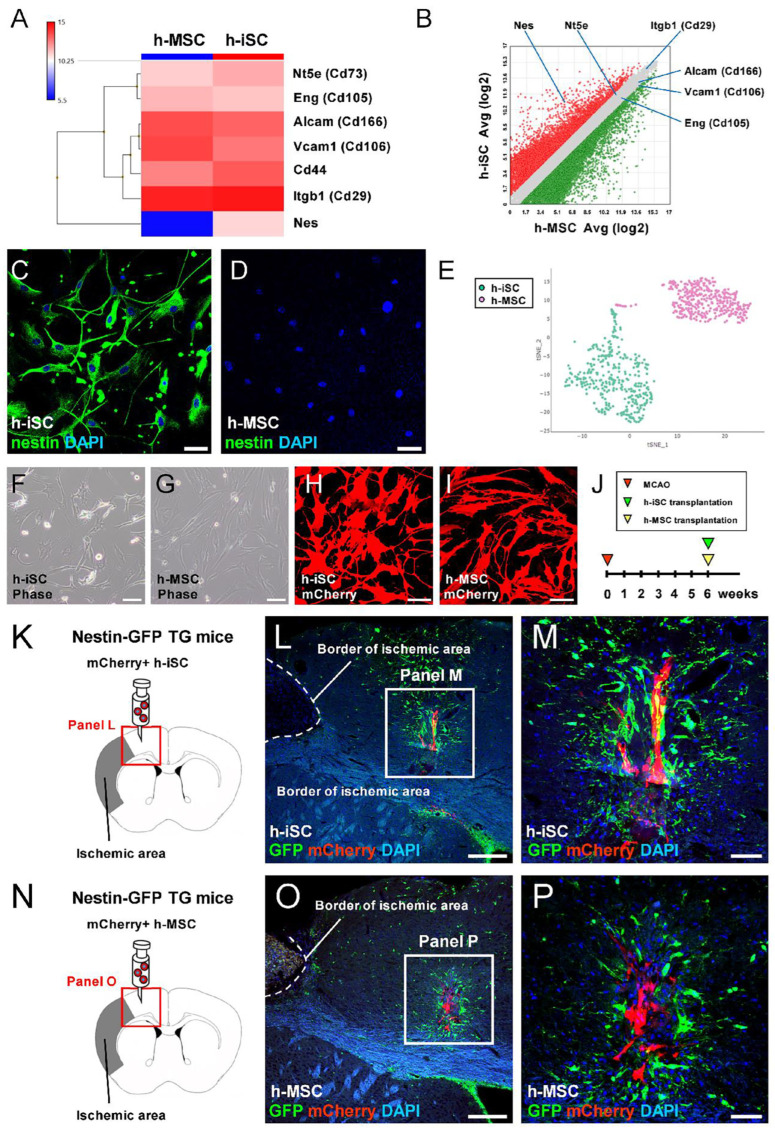
(**A**,**B**) Heatmap (**A**) and scatter plot (**B**) analyses of h-iSCs and h-MSCs. (**C**,**D**) h-iSCs (**C**) and h-MSCs (**D**) were immunostained with nestin (nestin [(**C**,**D**): green] and DAPI [(**C**,**D**): blue]). (**E**) scRNA-seq analysis of h-iSCs and h-MSCs. (**F**–**J**) h-iSCs (**F**) or h-MSCs (**G**) were labeled with mCherry. Then, mCherry^+^ h-iSCs (**H**) or h-MSCs (**I**) were grafted 6 weeks after MCAO (**J**). (**K**–**M**) mCherry^+^ h-iSCs were transplanted around the ischemic areas of nestin-GFP transgenic mice. Immunohistochemistry 3 days after transplantation showed that in addition to the GFP^+^ cells in the SVZ, several GFP^+^ NSPCs were located around the grafted mCherry^+^ h-iSCs (GFP [(**L**,**M**): green], mCherry [(**L**,**M**): red], and DAPI [**L**,**M**: blue]). (**N**–**P**) mCherry^+^ h-MSCs were transplanted around the ischemic areas of nestin-GFP transgenic mice. Immunohistochemistry 3 days after transplantation showed that in addition to the GFP^+^ cells in the SVZ, several GFP^+^ NSPCs were located around the grafted mCherry^+^ h-MSCs (GFP [(**O**,**P**): green], mCherry [(**O**,**P**): red], and DAPI [(**O**,**P**): blue]). Scale bars: 50 µm (**C**,**D**), 100 µm (**F**–**I**), 200 µm (**L**,**O**), and 50 µm (**M**,**P**). Abbreviations: DAPI, 4′,6-diamidino-2-phenylindole; GFP, green fluorescent protein; iSC, injury/ischemia-induced stem cell; MSC, mesenchymal stem cell; MCAO, middle cerebral artery occlusion; NSPC, neural stem/progenitor cell; scRNA-seq, single-cell RNA sequencing; SVZ, subventricular zone.

Based on a previous study, h-iSCs and h-MSCs are similar in terms of multipotency [8]. However, they are a distinct stem cell population: h-iSCs exhibit both neural and mesenchymal lineage markers; meanwhile, h-MSCs exhibit mesenchymal markers [8]. Consistent with this report, heat mapping analysis (Figure 1A) and scatter plot analysis (Figure 1B) using microarray showed that, although h-iSCs and h-MSCs expressed various mesenchymal lineage markers (*Nt5e*, *Eng*, *Alcam*, *Vcam1*, *Cd44*, and *Itgb1*), h-iSCs expressed neural lineage markers (*Nes*). Further, immunohistochemistry showed that h-iSCs (Figure 1C), but not h-MSCs (Figure 1D), highly expressed nestin. In addition, single-cell RNA sequencing (scRNA-seq) analysis revealed that the clustering of h-iSCs and h-MSCs is distributed without overlapping each other (Figure 1E). Hence, this finding confirms that they are different stem cell populations.

In a recent study, not only h-iSC transplantation [9] but also h-MSC transplantation [18] activated locally derived endogenous NSPCs. Therefore, h-iSCs (Figure 1F) and h-MSCs (Figure 1G) were initially labeled with an mCherry-carrying lentivirus vector. Then, the same numbers of mCherry^+^ h-iSCs (Figure 1H, 5.0 × 10^4^ cells/μL) or mCherry^+^ h-MSCs (Figure 1I, 5.0 × 10^4^ cells/μL) were transplanted into the post-stroke mouse brains of nestin-GFP transgenic mice 6 weeks after MCAO (Figure 1J). Consistent with previous reports [9,18], immunohistochemistry of the brain sections showed that several GFP^+^ cells were observed around the mCherry^+^ grafted sites both after h-iSC transplantation (Figure 1K–M) and h-MSC transplantation (Figure 1N–P). Hence, not only h-iSC transplantation but also h-MSC transplantation activated endogenous regionally derived NSPCs.

### 2.2. h-iSC Transplantation Exhibits a Higher Neurological Functional Improvement Compared with h-MSC Transplantation

**Figure 2:**
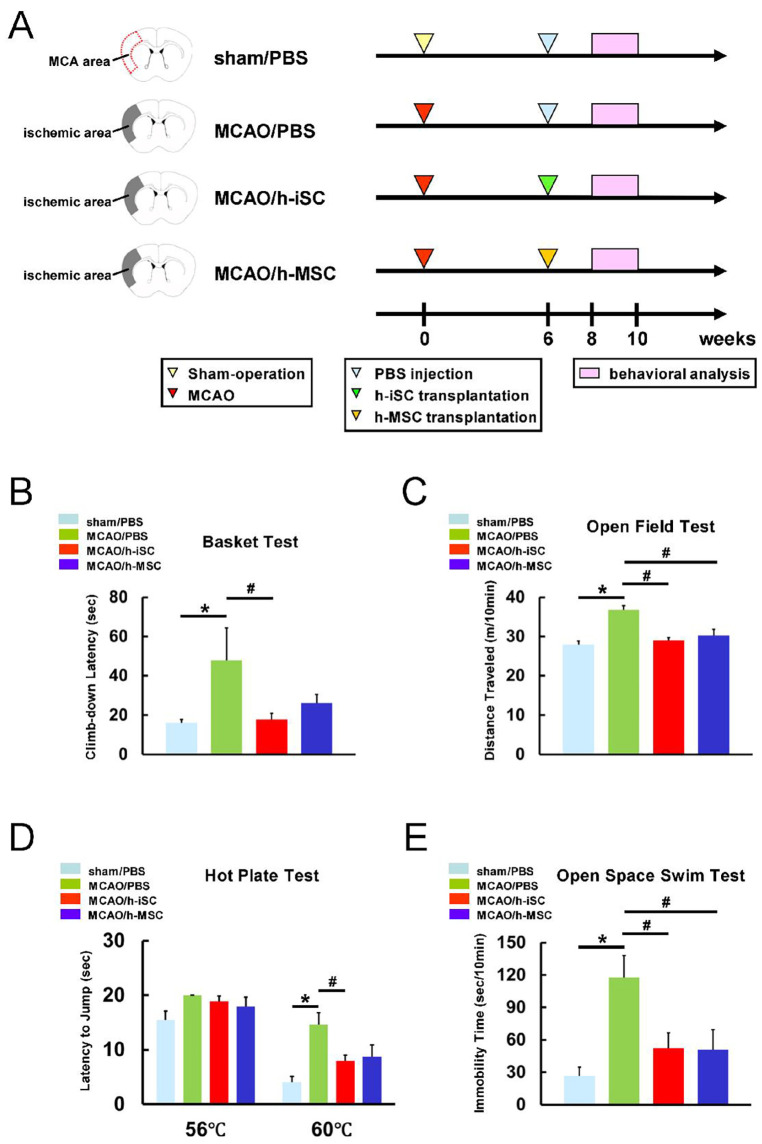
(**A**) Behavioral tests were performed on the four groups: (1) mice injected with PBS after a sham operation (sham/PBS group, *n* = 12), (2) mice injected with PBS after MCAO (MCAO/PBS group, *n* = 8), (3) mice administered h-iSCs after MCAO (MCAO/h-iSC group, *n* = 12), and (4) mice administered h-MSCs after MCAO (MCAO/h-MSC group, *n* = 10). (**B**–**E**) Performance levels in the basket (**B**), open-field (**C**), hot plate (**D**), and open-space swim (**E**) tests among the four groups. * *p* < 0.05 between the sham/PBS group (**B**–**E**). # *p* < 0.05 between the MCAO/PBS group (**B**–**E**). Abbreviations: iSC, injury/ischemia-induced stem cell; MCA, middle cerebral artery; MCAO, middle cerebral artery occlusion; MSC, mesenchymal stem cell; PBS, phosphate-buffered saline.

Next, h-iSCs (5.0 × 10^4^ cells/μL) and h-MSCs (5.0 × 10^4^ cells/μL) were transcranially transplanted into post-stroke mouse brains 6 weeks after MCAO, and the efficacy of these two different cell sources was compared using multiple tasks (Figure 2A). To facilitate the evaluation, the mice were divided into four groups, which were as follows: (1) mice injected with phosphate-buffered saline (PBS) after a sham operation (sham/PBS group), (2) mice injected with PBS after MCAO (MCAO/PBS group), (3) mice administered h-iSCs after MCAO (MCAO/h-iSC group), and (4) mice administered h-MSCs after MCAO (MCAO/h-MSC group) (Figure 2A).

The basket test was performed to assess motor function (Figure 2B). Results showed that the MCAO/PBS group had a significantly longer latency to reach the floor than the sham/PBS group. However, the latency to reach the floor was significantly shorter in the MCAO/h-iSC group than in the MCAO/PBS group. In contrast, it was not significantly shorter than that in the MCAO/h-MSC group. Based on these results, post-stroke mice experienced improvement in motor function after h-iSC transplantation.

The open-field test was performed to examine spontaneous locomotor activity (Figure 2C). The MCAO/PBS group had a significantly higher locomotor activity than the sham/PBS group. Hence, as stroke patients frequently displayed clinically [19], the mice presented with stroke-associated hyperactivity after MCAO. Nevertheless, the MCAO/h-iSC and MCAO/h-MSC groups had a significantly lower locomotor activity than the MCAO/PBS group. Therefore, stroke-associated hyperactivity was suppressed by cell transplantation.

The hot plate test was conducted to investigate the sensitivity of thermal nociception (Figure 2D). The latency to jump at 56 °C did not significantly differ among the four groups. The MCAO/PBS group had a significantly longer latency to jump at 60 °C than the sham/PBS group. Thus, the mice presented with stroke-associated thermal hypoalgesia after MCAO induction. However, the latency to jump at 60 °C was significantly shorter in the MCAO/h-iSC group than in the MCAO/PBS group. By contrast, it was not significantly shorter than that in the MCAO/h-MSC group. Therefore, h-iSC transplantation improved thermal hypoalgesia.

The open-space swim test was performed to assess the presence of depression-like symptoms (Figure 2E). The MCAO/PBS group had a significantly longer immobility time than the sham/PBS group. However, the MCAO/h-iSC and MCAO/h-MSC groups had a significantly shorter immobility time than the MCAO/PBS group. Thus, cell transplantation improved stroke-associated depression-like symptoms.

### 2.3. h-iSCs Have a Higher Effect on Increasing the Numbers of Endogenous NSPCs than h-MSCs Based on the Coculture Experiment

**Figure 3:**
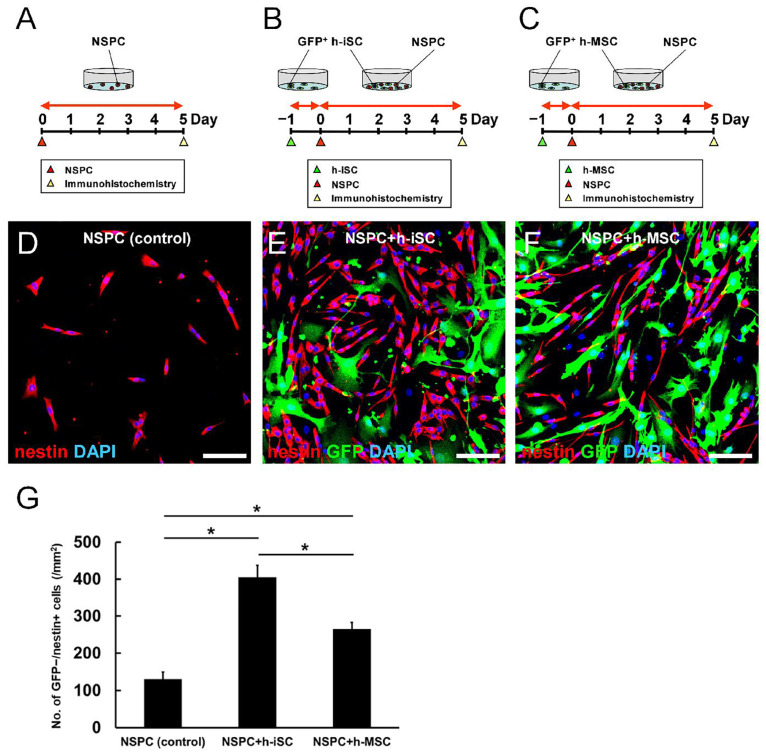
(**A**–**C**) Regionally derived NSPCs from post-stroke mouse brains were incubated alone (**A**) or cocultured with GFP^+^ h-iSCs (**B**) or GFP^+^ h-MSCs (**C**) for immunohistochemistry. (**D**–**G**) Immunohistochemistry showed that the numbers of nestin^+^ NSPCs (GFP^−^/nestin^+^ cells) in samples cocultured with h-iSCs (**E**) and h-MSCs (**F**) were significantly higher than those in samples cocultured with NSPCs alone (**D**). However, the numbers of nestin^+^ NSPCs in samples cocultured with h-iSCs were significantly higher than those in samples cocultured with h-MSCs (**G**) (nestin [(**D**–**F**): red], GFP [(**E**,**F**): green], and DAPI [(**D**–**F**): blue]). Scale bars: 100 µm (**D**–**F**). * *p* < 0.05 between the groups (**G**). *n* = 3 (12 data points) for each group (**G**). Abbreviations: DAPI, 4′,6-diamidino-2-phenylindole; GFP, green fluorescent protein; iSC, injury/ischemia-induced stem cell; MSC, mesenchymal stem cell; NSPC, neural stem/progenitor cell.

Thus far, our data showed that not only h-iSC transplantation but also h-MSC transplantation activated endogenous NSPCs around the grafted sites. Nevertheless, h-iSC transplantation was associated with a higher neurological functional improvement compared with h-MSC transplantation. To investigate the underlying mechanism, regionally derived NSPCs obtained from post-stroke mouse brains were incubated alone (Figure 3A) or cocultured with GFP^+^ h-iSCs (Figure 3B) or GFP^+^ h-MSCs (Figure 3C) for immunohistochemistry.

On day 5 after coincubation, the cells were fixed and subjected to immunohistochemistry with an antibody against nestin. Results showed that compared with NSPCs alone (Figure 3D,G), the numbers of nestin^+^ NSPCs (GFP^−^ and nestin^+^ cells [GFP^−^/nestin^+^ cells]) in cocultures with h-iSCs (Figure 3E,G) and h-MSCs (Figure 3F,G) were significantly higher. These results support the notion that h-iSCs or h-MSCs activated endogenous NSPCs (Figure 1K–P).

**Figure 4:**
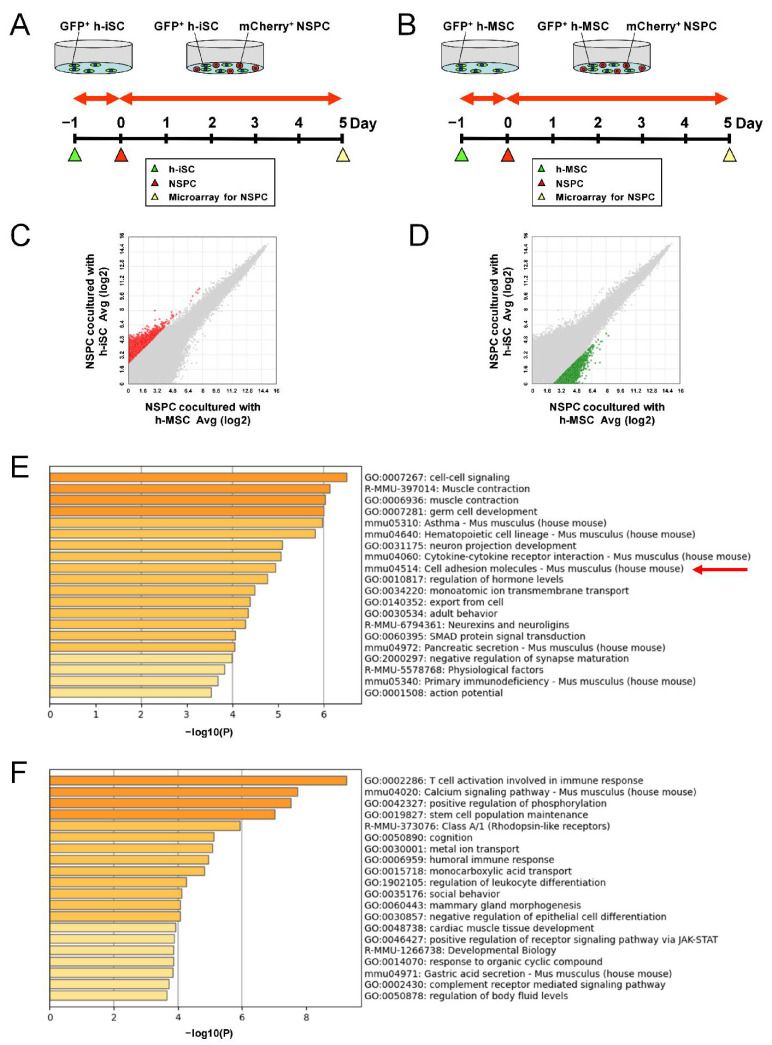
(**A**,**B**) NSPCs were cocultured with h-iSCs (**A**) or h-MSCs (**B**) for microarray analysis. (**C**,**D**) Scatter plots showing the distribution of genes upregulated more than 5-fold in NSPCs cocultured with h-iSCs relative to NSPCs cocultured with h-MSCs ((**C**), red plots) or genes upregulated more than 5-fold in NSPCs cocultured with h-MSCs relative to NSPCs cocultured with h-iSCs ((**D**), green plots). (**E**,**F**) List of the top 20 categories for genes overexpressed in NSPCs cocultured with h-iSCs (**E**) and NSPCs cocultured with h-MSCs (**F**) based on GO analysis. The former (**E**) included genes categorized in the “Cell adhesion molecules” category (a red arrow).

To investigate the underlying mechanism, microarray analysis was performed using NSPCs alone, NSPCs cocultured with h-iSCs, or NSPCs cocultured with h-MSCs. NSPCs cocultured with h-iSCs (Figure 4A) or h-MSCs (Figure 4B) were selectively collected via fluorescence-activated cell sorting (FACS), and their traits were analyzed. The gene expression levels that were significantly different between groups (>2-fold or <−2-fold) were subjected to pathway analysis.

We analyzed the cell cycle-related pathway and found that, compared with NSPCs alone, various cell cycle-related genes were upregulated in NSPCs cocultured with h-iSCs (Supplementary Figure S1) and h-MSCs (Supplementary Figure S2). However, gene expression patterns were not obviously different between NSPCs cocultured with h-iSCs and those cocultured with h-MSCs (Supplementary Figure S3).

We further investigated pathway analysis related to apoptosis. The results showed that, although various apoptosis-related genes were downregulated in NSPCs cocultured with h-iSCs (Supplementary Figure S4) and h-MSCs (Supplementary Figure S5), gene expression patterns were not obviously different between NSPCs cocultured with h-iSCs and those cocultured with h-MSCs (Supplementary Figure S6).

Therefore, although a higher effect on increasing the numbers of endogenous NSPCs by iSCs and h-MSCs is in part attributed to the upregulation of cell cycle and/or downregulation of apoptosis, a higher effect on increasing the numbers of endogenous NSPCs by h-iSCs than h-MSCs is likely derived from other mechanisms.

To investigate the underlying mechanism, the genes that were expressed significantly higher in NSPCs cocultured with h-iSCs than in those cocultured with h-MSCs (>5-fold) (Figure 4C) and the genes that were expressed significantly lower in NSPCs cocultured with h-iSCs than in those cocultured with h-MSCs (<−5-fold)] (Figure 4D) were analyzed via gene ontology (GO) analysis. A list of the top 20 categories obtained from the former (Figure 4E) and the latter analysis (Figure 4F) was shown. The results showed that the term “Cell adhesion molecules” was present in the list in Figure 4E, while this term was not present in the list in Figure 4F. These results suggest that a higher effect of increasing the numbers of endogenous NSPCs by h-iSCs than h-MSCs is in part ascribed to the regulation of cell adhesion molecules, which can affect the fate of NSPCs [20].

### 2.4. The Genes Related to Neuronal Lineages Are Enriched in NSPCs After Coincubation with h-iSCs Compared with Those After Coincubation with h-MSCs

**Figure 5:**
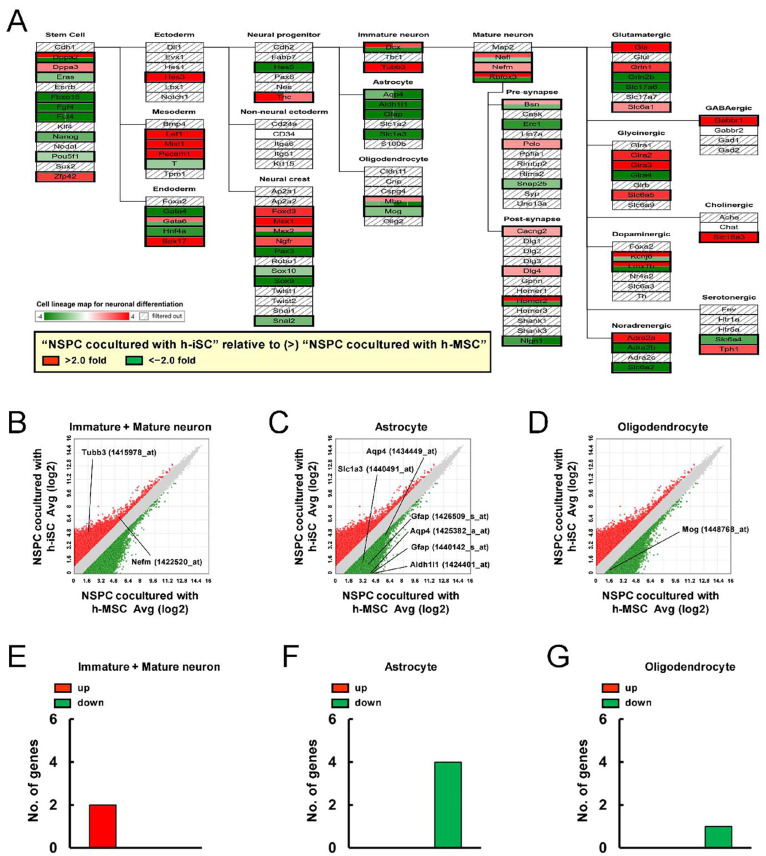
(**A**) Pathway analysis of the cell lineage map for neuronal differentiation showed that the expression of various neural-lineage-related genes in NSPCs after coincubation with h-iSCs was significantly upregulated (2-fold higher, red box) and/or downregulated (2-fold lower, green box) compared with that in NSPCs after coincubation with h-MSCs. (**B**–**D**) The scatter plot analysis showed the distribution of significantly upregulated (2-fold higher, red plots) and/or downregulated (2-fold lower, green plots) genes subcategorized into “Immature neuron” and “Mature neuron” (**B**), “Astrocyte” (**C**), and “Oligodendrocyte” (**D**) in the cell lineage map for neuronal differentiation. (**E**–**G**) The numbers of significantly upregulated (2-fold higher, red box) and/or downregulated (2-fold lower, green box) genes subcategorized as “Immature neuron” and “Mature neuron” (**E**), “Astrocyte” (**F**), and “Oligodendrocyte” (**G**) in the cell lineage map for neuronal differentiation. Abbreviations: iSC, injury/ischemia-induced stem cell; MSC, mesenchymal stem cell; NSPC, neural stem/progenitor cell.

The phenotypic change in NSPCs was investigated in more detail. The genes that were expressed significantly higher in NSPCs cocultured with h-iSCs than in those cocultured with h-MSCs (>2-fold) and the genes that were expressed significantly lower in NSPCs cocultured with h-iSCs than in those cocultured with h-MSCs (<−2-fold) were analyzed according to category in the cell lineage map for neuronal differentiation via pathway analysis (Figure 5A).

Results showed that the genes related to neuronal lineages, such as “Immature neuron” (*Tubb3*) and “Mature neuron” (*Nefm*), in NSPCs after coincubation with h-iSCs were enriched compared with those after coincubation with h-MSCs (Figure 5A,B,E). In contrast, the expression of genes related to astrocytic (“Astrocyte”: *Aqp4*, *Aldh1l1*, *Gfap*, and *Slc1a3*) (Figure 5A,C,F) and oligodendrocytic lineages (“Oligodendrocyte”: *Mog*) (Figure 5A,D,G) in NSPCs after coincubation with h-iSCs was downregulated compared with that in NSPCs after coincubation with h-MSCs. Hence, the presence of h-iSCs, compared with that of h-MSCs, can possibly promote the differentiation toward neuronal lineages rather than glial lineages against NSPCs.

### 2.5. Comparative Analysis of the Gene Profiles of h-iSCs Between h-iSC Monocultures and h-iSCs Cocultured with NSPCs

**Figure 6:**
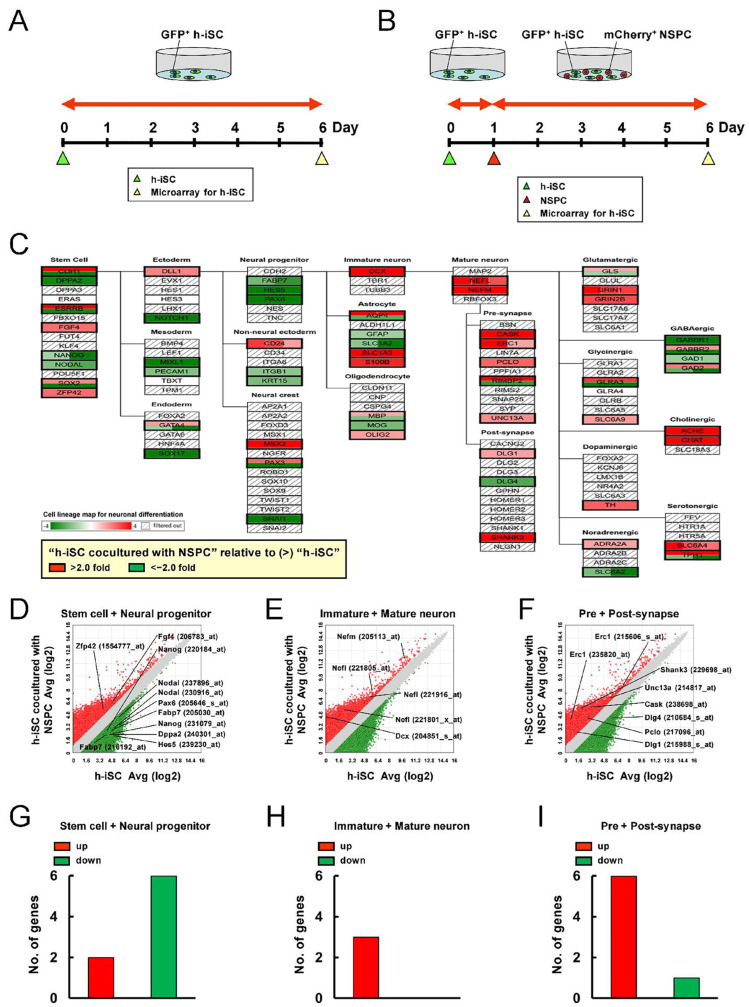
(**A**,**B**) h-iSCs were cultured alone (**A**) or cocultured with NSPCs (**B**) for microarray analysis. (**C**) Pathway analysis of the cell lineage map for neuronal differentiation showed that the expression of various neural lineage-related genes in h-iSCs after coincubation with NSPCs was significantly upregulated (2-fold higher, red box) and/or downregulated (2-fold lower, green box) compared with those in h-iSCs alone. (**D**–**F**) The scatter plot analysis showed the distribution of significantly upregulated (2-fold higher, red plots) and/or downregulated (2-fold lower, green plots) genes subcategorized into “Stem cell” and “Neural progenitor” (**D**), “Immature neuron” and “Mature neuron” (**E**), and “Pre-synapse” and “Post-synapse” (**F**) in the cell lineage map for neuronal differentiation. (**G**–**I**) The numbers of significantly upregulated (2-fold higher, red box) and/or downregulated (2-fold lower, green box) genes subcategorized into “Stem cell” and “Neural progenitor” (**G**), “Immature neuron” and “Mature neuron” (**H**), and “Pre-synapse” and “Post-synapse” (**I**) in the cell lineage map for neuronal differentiation. Abbreviations: iSC, injury/ischemia-induced stem cell; NSPC, neural stem/progenitor cell.

Next, the effect of the presence of NSPCs on the fates of neural lineages in h-iSCs or h-MSCs was investigated. The gene expression profiles of h-iSCs between h-iSC monocultures (Figure 6A) and h-iSCs cocultured with NSPCs (Figure 6B) were initially compared via microarray analysis. The genes that were expressed significantly higher in h-iSCs cocultured with NSPCs relative to those in h-iSC monocultures (>2-fold) and the genes that were expressed significantly lower in h-iSCs cocultured with NSPCs relative to those in h-iSC monocultures (<−2-fold) were analyzed according to the category in the cell lineage map for neuronal differentiation via pathway analysis (Figure 6C).

Results showed that the expression of some genes related to “Stem cell” (*Fgf4*, *Zep42*) was more upregulated after coincubation with NSPCs (Figure 6C,D,G). However, the expression of a larger number of genes related to “Stem cell” (*Dppa2*, *Nanog*, and *Nodal*) and “Neural progenitor” (*Fabp7*, *Hes5*, and *Pax6*) was downregulated after coincubation with NSPCs (Figure 6C,D,G). This suggests that h-iSCs lost their stemness after cell–cell interaction with NSPCs. In support of this notion, the expression of genes related to “Immature neuron” (*Dcx*) and “Mature neuron” (*Nefl*, *Nefm*) was upregulated in h-iSCs after coincubation with NSPCs relative to h-iSCs alone (Figure 6C,E,H). Similarly, the expression of several genes related to “Pre-synapse” (*Cask*, *Erc1*, *Pclo*, and *Unc13a*) and “Post-synapse” (*Dlg1*, *Shank3*) was upregulated in h-iSCs after coincubation with NSPCs (Figure 6C,F,I).

### 2.6. Comparative Analysis of the Gene Profiles of h-MSCs Between h-MSC Monocultures and h-MSCs Cocultured with NSPCs

**Figure 7:**
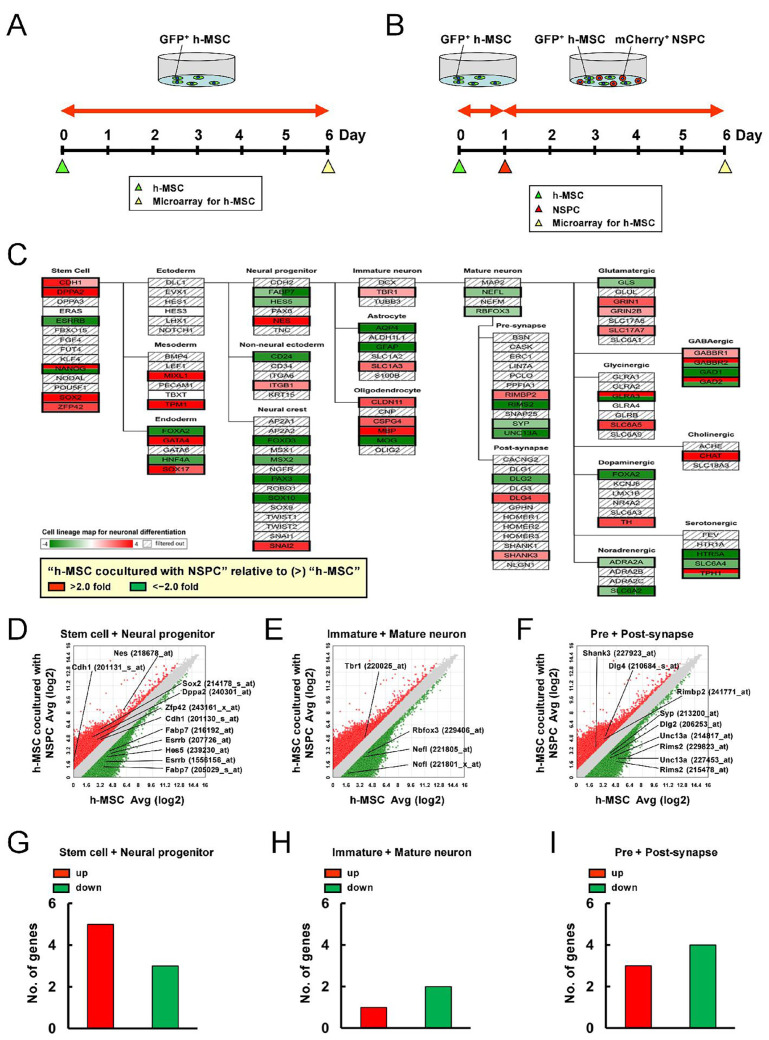
(**A**,**B**) h-MSCs were cultured alone (**A**) or cocultured with NSPCs (**B**) for microarray analysis. (**C**) Pathway analysis of the cell lineage map for neuronal differentiation showed that the expression of various neural lineage-related genes in h-MSCs after coincubation with NSPCs was significantly upregulated (2-fold higher, red box) and/or downregulated (2-fold lower, green box) compared with that in h-MSCs alone. (**D**–**F**) The scatter plot analysis showed the distribution of significantly upregulated (2-fold higher, red plots) and/or downregulated (2-fold lower, green plots) genes subcategorized into “Stem cell” and “Neural progenitor” (**D**), “Immature neuron” and “Mature neuron” (**E**), and “Pre-synapse” and “Post-synapse” (**F**) in the cell lineage map for neuronal differentiation. (**G**–**I**) The numbers of significantly upregulated (2-fold higher, red box) and/or downregulated (2-fold lower, green box) genes subcategorized into “Stem cell” and “Neural progenitor” (**G**), “Immature neuron” and “Mature neuron” (**H**), and “Pre-synapse” and “Post-synapse” (**I**) in the cell lineage map for neuronal differentiation. Abbreviations: MSC, mesenchymal stem cell; NSPC, neural stem/progenitor cell.

The gene expression profiles of h-MSCs between h-MSC monocultures (Figure 7A) and h-MSCs cocultured with NSPCs (Figure 7B) were compared via microarray analysis. The genes that were expressed significantly higher in h-MSCs cocultured with NSPCs relative to those in h-MSC monocultures (>2-fold) and the genes that were expressed significantly lower in h-MSCs cocultured with NSPCs relative to those in h-MSC monocultures (<−2-fold) were analyzed according to category in the cell lineage map for neuronal differentiation via pathway analysis (Figure 7C).

Results showed that the expression of genes related to “Stem cell” (*Cdh1*, *Dppa2*, *Sox2*, and *Zep42*) and “Neural progenitor” (*Nes*) was more upregulated after coincubation with NSPCs (Figure 7C,D,G). In contrast, a fewer number of genes related to “Stem cell” (*Esrrb*) and “Neural progenitor” (*Fabp7*, *Hes5*) had downregulated expression after coincubation with NSPCs (Figure 7C,D,G). Hence, h-MSCs maintained their stemness after cell–cell interactions with NSPCs. In support of this notion, a larger number of genes related to neurons (“Mature neuron”: *Nefl*, *Rbfox3*) had downregulated expression in h-MSCs after coincubation with NSPCs than after h-MSC monoculture (Figure 7C,E,H). Similarly, a larger number of genes related to synapse (“Pre-synapse”: *Rims2*, *Syp*, and *Unc13a* and “Post-synapse”: *Dlg2*) had downregulated expression in h-MSCs after coincubation with NSPCs compared with after h-MSC monoculture (Figure 7C,F,I).

### 2.7. Enrichment of Gene Expression Related to Synapse Formation and Neurotransmitter-Releasing Neurons in h-iSCs After Coincubation with NSPCs Based on a Comparative Analysis of h-iSCs and h-MSCs

**Figure 8:**
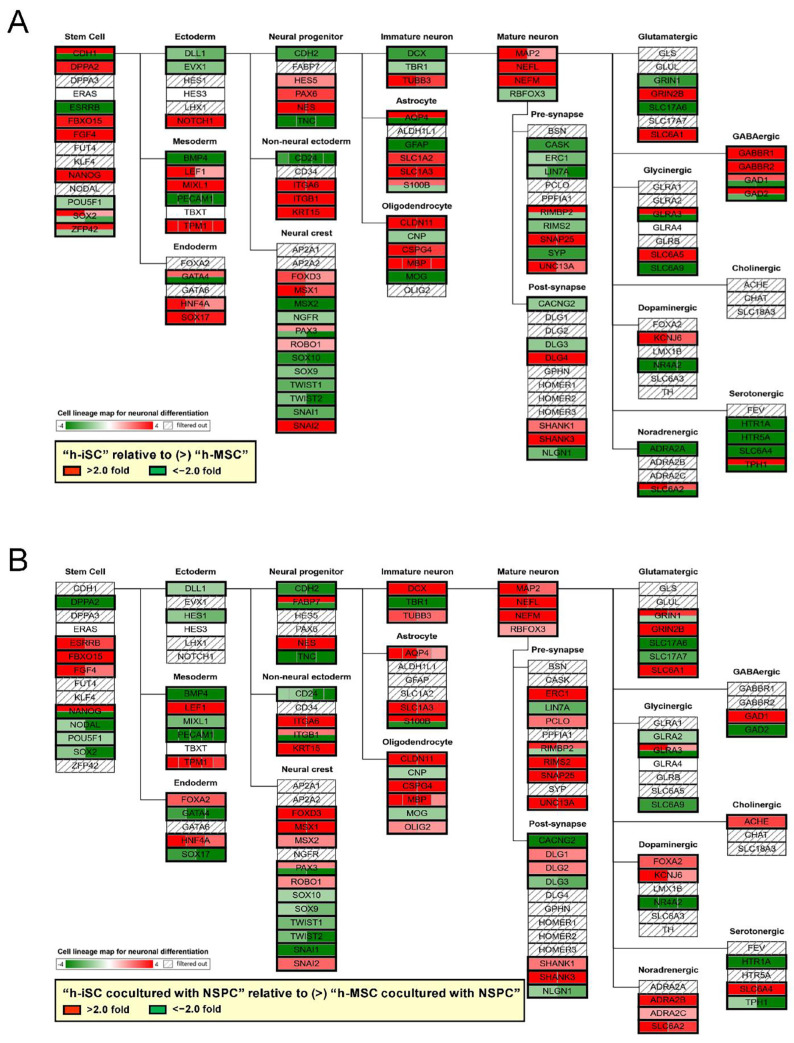
(**A**,**B**) Pathway analysis of the cell lineage map for neuronal differentiation was performed between h-iSCs and h-MSCs before (**A**) and after coincubation with NSPCs (**B**). The expression of neural lineage-related genes in h-iSCs relative to those in h-MSCs was significantly upregulated (2-fold higher, red box) and/or downregulated (2-fold lower, green box). Abbreviations: iSC, injury/ischemia-induced stem cells; MSC, mesenchymal stem cell; NSPC, neural stem/progenitor cell.

Thus far, our data showed that the presence of NSPCs promoted the neuronal differentiation in h-iSCs. Meanwhile, the presence of NSPCs maintained stemness in h-MSCs. Therefore, the genes that were expressed significantly higher in h-iSCs relative to those in h-MSCs (>2-fold) and the genes that were expressed significantly lower in h-iSCs relative to those in h-MSCs (<−2-fold) were analyzed according to the category in the cell lineage map for neuronal differentiation via pathway analysis. Then, the gene expression patterns of h-iSCs relative to those of h-MSCs before (Figure 8A) and after coincubation with NSPCs were compared (Figure 8B).

**Figure 9:**
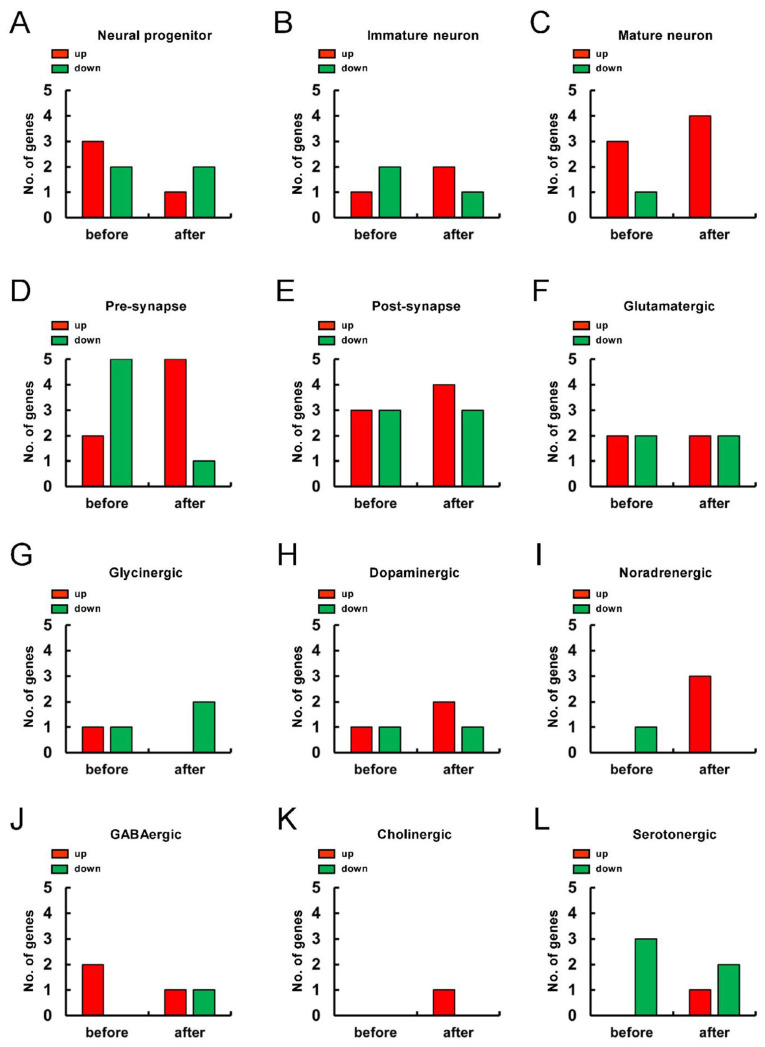
(**A**–**L**) Pathway analysis of the cell lineage map for neuronal differentiation was performed between h-iSCs and h-MSCs before and after coincubation with NSPCs. The expression of significantly upregulated (2-fold higher, red box) and/or downregulated (2-fold lower, green box) genes in h-iSCs relative to those in h-MSCs, which were subcategorized into “Neural progenitor” (**A**), “Immature neuron” (**B**), “Mature neuron” (**C**), “Pre-synapse” (**D**), “Post-synapse” (**E**), “Glutamatergic” (**F**), “Glycinergic” (**G**), “Dopaminergic” (**H**), “Noradrenergic” (**I**), “GABAergic” (**J**), “Cholinergic” (**K**), and “Serotonergic” (**L**) in the cell lineage map for neuronal differentiation, are presented. Abbreviations: iSC, injury/ischemia-induced stem cell; MSC, mesenchymal stem cell; NSPC, neural stem/progenitor cell.

The numbers of upregulated genes (>2-fold) or downregulated genes (<−2-fold) in the “Neural progenitor” (Figure 9A), “Immature neuron” (Figure 9B), “Mature neuron” (Figure 9C), “Pre-synapse” (Figure 9D), “Post-synapse” (Figure 9E), “Glutamatergic” (Figure 9F),“Glycinergic” (Figure 9G),“Dopaminergic” (Figure 9H),“Noradrenergic” (Figure 9I), “GABAergic” (Figure 9J), “Cholinergic” (Figure 9K), and “Serotonergic” (Figure 9L) categories before and after coincubation with NSPCs are presented.

Results showed that the expression of genes related to “Neural progenitor” was downregulated after coincubation with NSPCs (Figure 9A). In contrast, the expression of genes related to neurons, such as “Immature neuron” (Figure 9B) and “Mature neuron” (Figure 9C), was upregulated after coincubation with NSPCs. In addition, the expression of genes related to “Pre-synapse” (Figure 9D) and “Post-synapse” (Figure 9E) was higher after coincubation with NSPCs. Notably, the expression level of genes that were related to neurotransmitter-producible neurons, such as “Dopaminergic” (Figure 9H), “Noradrenergic” (Figure 9I), and “Serotonergic” (Figure 9L) neurons, was more upregulated after coincubation with NSPCs. Therefore, NSPCs likely promoted transdifferentiation toward functional neurons predominately in h-iSCs.

### 2.8. Formation of Higher Numbers of Neuronal Networks with the Coexistence of NSPCs and h-iSCs

Thus far, our data showed that the NSPC-derived expression of mature neurons is accelerated by the presence of h-iSCs rather than h-MSCs. In turn, NSPCs promoted the acquisition of neuronal traits in h-iSCs compared with h-MSCs. Therefore, compared with the coexistence of NSPCs and h-MSCs, the coexistence of NSPCs and h-iSCs can lead to the formation higher numbers of neuronal networks.

**Figure 10:**
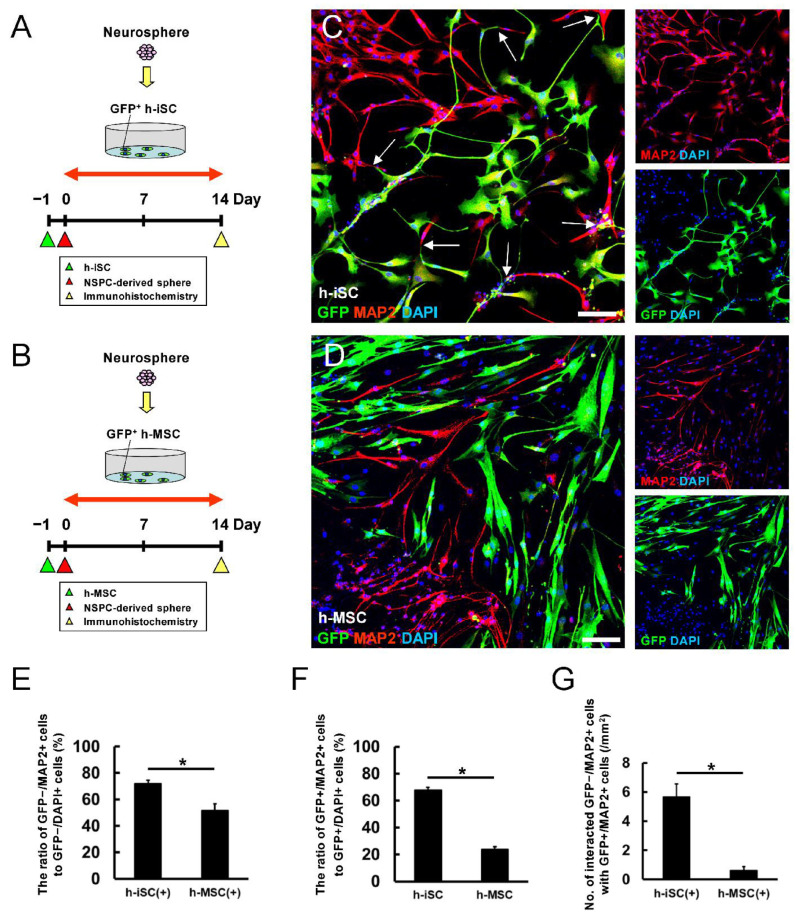
(**A**,**B**) GFP^+^ h-iSCs (**A**) or h-MSCs (**B**) were cocultured with NSPC-derived neurospheres for 2 weeks. (**C**,**D**) Immunocytochemistry of NSPC-derived neurons (GFP^−^/MAP2^+^ cells) cocultured with h-iSCs (**C**) and h-MSCs (**D**) (MAP2 [(**C**,**D**): red], GFP [(**C**,**D**): green], and DAPI [(**C**,**D**): blue]). (**E**) The ratio of NSPC-derived neurons (GFP^−^/MAP2^+^ cells to GFP^−^/DAPI^+^ cells) after coculture with h-iSCs was higher than that after coculture with h-MSCs. (**F**) The ratio of h-iSC-derived neurons (GFP^+^/MAP2^+^ cells to GFP^+^/DAPI^+^ cells) was significantly higher than that of h-MSC-derived neurons (GFP^+^/MAP2^+^ cells to GFP^+^/DAPI^+^ cells) in the presence of NSPCs. (**G**) The number of NSPC-derived neurons (GFP^−^/MAP2^+^ cells) that interacted with h-iSC-derived neurons (GFP^+^/MAP2^+^ cells) was significantly higher than that of NSPC-derived neurons that interacted with h-MSC-derived neurons (GFP^+^/MAP2^+^ cells). Scale bars: 100 µm (**C**,**D**). * *p* < 0.05 between the h-iSC(+) and h-MSC(+) groups (**E**,**G**). *p* < 0.05 between the h-iSC and h-MSC groups (**F**). *n* = 3 (nine data points) for each group (**E**–**G**). Abbreviations: DAPI, 4′,6-diamidino-2-phenylindole; GFP, green fluorescent protein; iSC, injury/ischemia-induced stem cells; NSPC, neural stem/progenitor cell; MAP2, microtubule-associated protein 2; MSC, mesenchymal stem cell.

To further elucidate this notion, GFP^+^ h-iSCs (Figure 10A) or GFP^+^ h-MSCs (Figure 10B) were plated onto dishes. After 1 day, NSPC-derived neurospheres were plated onto each dish and further cocultured for 2 weeks. Then, the cells were fixed and immunostained with an antibody against the mature neuronal marker MAP2. Immunohistochemistry showed that the population of NSPC-derived MAP2^+^ neurons (GFP^−^/MAP2^+^ cells) in the presence of h-iSCs (Figure 10C,E) was significantly higher than that in the presence of h-MSCs (Figure 10D,E).

In the presence of NSPCs, h-iSC-derived cells (GFP^+^ cells) frequently produced neurite-like formations and differentiated into MAP2^+^ mature neurons (GFP^+^/MAP2^+^ cells) (Figure 10C). In addition, the population of h-iSC-derived MAP2^+^ neurons (GFP^+^/MAP2^+^ cells) was significantly higher than that of h-MSC-derived MAP2^+^ neurons (GFP^+^/MAP2^+^ cells) (Figure 10F).

Notably, h-iSC-derived neurons (GFP^+^/MAP2^+^ cells) and NSPC-derived neurons (GFP^−^/MAP2^+^ cells) frequently interacted with each other (Figure 10C, arrows), and the number of interactions between NSPC-derived neurons (GFP^−^/MAP2^+^ cells) and h-iSC-derived neurons (GFP^+^/MAP2^+^ cells) was significantly higher than that between NSPC-derived neurons (GFP^−^/MAP2^+^ cells) and h-MSC-derived neurons (GFP^+^/MAP2^+^ cells) (Figure 10G).

## 3. Discussion

To the best of our knowledge, this study first compared the efficacy of cell transplantation between h-iSCs and h-MSCs in mice after ischemic stroke. h-iSC transplantation was found to be associated with a higher neurological functional improvement than M-iSC transplantation. Hence, h-iSCs can be a novel source of cell therapy for ischemic stroke.

The actual traits of h-iSCs remain unclear. However, a previous study showed that h-iSCs are unique stem cell populations that have both neural and mesenchymal markers [8]. In addition, both h-iSCs and h-MSCs differentiated into mesenchymal lineages (e.g., adipocytes, osteoblast, and chondrocytes) in vitro. Nevertheless, their potential to differentiate into mesenchymal lineages was higher in h-MSCs than in h-iSCs. In contrast, their potential to differentiate into neuronal lineages was higher in h-iSCs than in h-MSCs in vitro [8]. Notably, using multielectrode arrays, h-iSCs were found to frequently differentiate into electrophysiologically functional neurons. Meanwhile, h-MSCs rarely did [8]. Based on these findings, h-iSCs have a higher neurogenic potential than h-MSCs. In support of this notion, a previous study showed that h-iSCs transplanted into post-stroke mice transdifferentiated into mature neurons and formed neuronal networks with endogenous neurons [9].

Increasing evidence has shown that MSCs rarely transdifferentiate into functional neurons [8,21,22]. Nevertheless, several studies, including preclinical studies, have revealed that MSC transplantation had positive effects via multiple mechanisms, including immunomodulatory effects [12,13], vasculogenesis [14], and MSC-derived factors (e.g., neurotrophic factors [15], extracellular vesicle [13,16], and microRNA [17]). Further, clinical trials have reported the usefulness of MSC transplantation [23,24,25,26,27]. Therefore, MSC transplantation can be a strong candidate of cell therapy targeting patients with stroke.

The current study compared the efficacy of h-iSC transplantation and h-MSC transplantation. Consistent with previous findings [9,18], h-iSC or h-MSC transplantation activated regionally derived endogenous NSPCs around transplanted sites. Occasionally, transplanted h-iSCs were also observed at the SVZ, suggesting that h-iSCs may favor this region. To investigate the effect of h-iSCs or h-MSCs on NSPCs in detail, using NSPCs extracted from brain ischemic areas, h-iSCs or h-MSCs were cocultured with NSPCs, and the gene expression was compared via microarray analysis. Results showed that the expression of neuronal lineage markers in NSPCs cocultured with h-iSCs was more upregulated than that in NSPCs cocultured with h-MSCs. In contrast, the expression of glial lineage markers in NSPCs cocultured with h-iSCs was more downregulated than that in NSPCs cocultured with h-MSCs. These findings were consistent with those of previous studies showing that MSCs promoted the glial differentiation of NSPCs [18,28,29]. Taken together, these results showed that h-iSCs have a higher neuronal differentiation potential against NSPCs than h-MSCs.

In this study, to investigate the effect of NSPCs on h-iSCs, h-iSCs alone were incubated or h-iSCs were cocultured with NSPCs. Then, the gene expression was compared via microarray analysis. Results showed that the expression of markers related to stem cell and neural progenitors was downregulated in h-iSCs in the presence of NSPCs. In contrast, the expression of markers related to neurons and synapse was upregulated in h-iSCs in the presence of NSPCs. Therefore, the presence of NSPCs promoted the shift from a stem cell state toward neuronal lineages against h-iSCs.

Moreover, to assess the effect of NSPCs on h-MSCs, h-MSCs alone were incubated or h-MSCs were cocultured with NSPCs. Next, the gene expression was compared via microarray analysis. Results showed that the expression of markers related to stem cell and neural progenitors was upregulated in h-MSCs in the presence of NSPCs. In contrast, the expression of markers related to neurons and synapses was downregulated in h-MSCs in the presence of NSPCs. Therefore, the presence of NSPCs promoted the shift from neuronal lineages toward a stem cell state against h-MSCs.

In the current study, the neurological functions of mice after h-iSC or h-MSC transplantation were compared. Consistent with previous studies on h-iSC [9] or h-MSC transplantation [18], not only h-iSC transplantation but also h-MSC transplantation improved neurological functions in various tasks. However, h-iSC transplantation had greater effects than h-MSC transplantation. Although the underlying mechanism remains unclear, the gene expression patterns of h-iSCs relative to those of h-MSCs showed that the expression of genes related to synapse, dopaminergic, noradrenergic, cholinergic, and serotonergic neurons was more upregulated after coincubation with NSPCs. Dopaminergic [30,31], noradrenergic [32,33], cholinergic [34], and serotonergic neurons [35,36] are crossly related to various brain functions, such as cognition, memory, mood, motivation, reward, addictive behaviors, and voluntary movement. Thus, the difference in functional improvement between h-iSC transplantation and h-MSC transplantation can be, in part, attributed to variations in the abovementioned results.

Moreover, microarray analysis showed that the expression of NSPC-derived neuronal-related genes in the presence of h-iSCs was more upregulated than that in the presence of h-MSCs. Therefore, the functional neuronal network formation between endogenous NSPC-derived neurons and grafted h-iSC-derived neurons is more likely to occur after h-iSC transplantation than after h-MSC transplantation. In support of this hypothesis, the current study showed that the number of MAP2^+^ mature neuronal networks between NSPC-derived neurons and h-iSC-derived neurons was significantly higher than those between NSPC-derived neurons and h-MSC-derived neurons based on coculture experiments. The precise traits of transplanted h-iSCs or h-MSCs should be elucidated in further studies. However, our previous study showed that some transplanted h-iSCs differentiated into MAP2^+^ mature neurons, which likely formed neural networks with endogenous MAP2^+^ mature neurons [9]. However, despite the use of immunodeficient mice, transplanted h-iSCs gradually decreased during several weeks [9]. Although we do not know the exact reason, the difference in species between the host and graft may affect this result. Therefore, the efficiency by cell transplantation and the fate of transplanted cells should be further evaluated by studies using the same species between the host and graft. Moreover, to identify the exact fate of endogenous NSPCs activated after cell transplantation, genetic fate-mapping studies using a marker for NSPCs would be needed. Nevertheless, future in vivo studies must be conducted to validate the hypothesis that a greater number of neuronal networks can be formed between NSPC-derived neurons and iSC-derived neurons.

The current study had several limitations. For example, we performed coculture experiments in medium that included 2% fetal bovine serum (FBS) because h-iSCs and NSPCs were maintained at this FBS concentration. However, h-MSCs were maintained in 10% FBS before coculture experiments. Therefore, culture conditions, such as the FBS concentration, might affect the fate of h-MSCs, thereby influencing the results of coculture experiments using h-MSCs. In this study, we used nestin-GFP transgenic mice to investigate the fate of endogenous NSPCs. However, nestin is expressed in various cell types, including pericytes [37] and endothelial cells [38], other than NSPCs. Therefore, an additional study using other transgenic mouse lines, such as Sox2-GFP transgenic mice [39], would be helpful to precisely investigate the fate of endogenous NSPCs.

Due to the advance of therapies for stroke patients, such as endovascular therapies, some patients can receive the treatment during acute periods. In contrast, no efficient therapies for chronic periods are available, while many stroke patients suffer from sequelae. Therefore, in this study, we investigated the efficacy of iSC transplantation in chronic periods. However, the efficacy of cell transplantation differs among the time points after ischemic stroke [40]. Moreover, a previous study showed that the fate of transplanted cells differs among the regions after ischemic stroke [41]. Therefore, the optimal time point and position regarding iSC transplantation should be elucidated in future studies.

Although we previously showed that iSCs derived from post-stroke mouse [7] and human brains [8] had the potential to differentiate into various cell types in vitro, it remains unclear whether they can exhibit multipotency in vivo. In addition, until now, we have not obtained the findings that transplanted iSCs differentiate into the undesirable cells, such as tumor cells. However, the safety of transplanted iSCs should be carefully investigated in further studies.

To proceed with the h-iSC research from preclinical trials to clinical trials, further issues should be resolved. For example, this study used BM-derived MSCs. However, other than from the bone marrow [42], MSCs are also available from various organs, such as adipose tissue [43] and the umbilical cord [28]. As the ability of MSCs varies among cell types [44], the advantage of h-iSCs should be investigated using different types of MSCs. Moreover, considering their clinical application, MSCs could be more accessible than iSCs. However, h-iSCs extracted from ischemic areas can be easily expanded in medium containing trophic factors, such as basic fibroblast growth factor (bFGF) and epidermal growth factor (EGF), and h-iSCs can maintain their properties even after thawing frozen cells. Therefore, h-iSCs expanded in vitro can be used as a source of exogenous cell transplantation. Alternatively, by identifying the traits of h-iSCs in more detail in future studies, h-iSCs might be created using iPS cells. Thus, h-iSCs derived from iPS cells can be used as a source for not only allogenic transplantation but also autologous cell transplantation.

## 4. Materials and Methods

### 4.1. Induction of Ischemic Stroke

The Animal Care Committee of Hyogo Medical University approved the experimental procedures (approval number: 18-074, 2019-10-3, and 22-019AG). Permanent focal cerebral ischemia was induced in 6–10-week-old adult mice (CB-17/Icr-scid/scid Jcl mice [Clea Japan Inc., Tokyo, Japan] or nestin-GFP TG mice [CB-17 background] that were produced by crossing B6.Cg-Tg(Nes-EGFP)1Yamm mice [RIKEN BioResource Research Center, Ibaraki, Japan] [45] and CB-17/Icr-+/+Jcl mice [Clea Japan Inc.] using backcrossing techniques, as described in previous studies) [6,9]. In brief, the mice were subjected to ligation and interruption of the distal portion of the left middle cerebral artery (MCA) under isoflurane anesthesia, and MCAO was induced, as described in previous studies [6,9]. This background of mice resulted in a highly reproducible ischemic stroke model with high survival rates, as described in a previous study [6].

### 4.2. Cell Transplantation

Putative h-iSCs were isolated from the human ischemic tissue samples, as described previously. In brief, post-stroke human brain samples were obtained from patients who satisfied the criteria, as described in a previous report [8]. Written informed consent was collected from all participants. Human tissue samples were treated in accordance with the Declaration of Helsinki, and the Ethics Committee of Hyogo Medical University approved the study protocol (approval numbers: 1776, 0385). Then, h-iSCs were maintained in Dulbecco’s Modified Eagle’s Medium (DMEM)/F12 (Thermo Fisher Scientific, Waltham, MA, USA) containing bFGF (20 ng/mL; PeproTech, Rocky Hill, NJ, USA), EGF (20 ng/mL; PeproTech), 1% N2 (Thermo Fisher Scientific), and 2% FBS and were used, as described in previous studies [9,18]. h-MSCs (PT-2501, Lonza, Basil, Switzerland) were maintained in the media, according to the manufacturer’s instructions, and were used, as described in previous studies [9,18].

h-iSCs or h-MSCs were transplanted into CB-17/Icr-scid/scid Jcl mice (immunodeficient mice), as described in previous studies [9,18]. Alternatively, h-iSCs (mCherry^+^ h-iSCs) or h-MSCs transfected with mCherry-expressing lentivirus vectors (mCherry^+^ h-MSCs) were transplanted into nestin-GFP TG mice, as described in previous studies [9,18]. Briefly, h-iSCs (0.5 μL; 1.0 × 10^5^ cells/μL), h-MSCs (0.5 μL; 1.0 × 10^5^ cells/μL), mCherry^+^ h-iSCs (0.5 μL; 1.0 × 10^5^ cells/μL), or mCherry^+^ h-MSCs (0.5 μL; 1.0 × 10^5^ cells/μL) were transcranially transplanted into the peri-ischemic areas (coordinates from bregma: anterior–posterior, 0 mm; medial–lateral, + 2.5 mm; and dorsal–ventral, −2.5 mm) 6 weeks after MCAO under isoflurane anesthesia. PBS (0.5 μL) was administered to control mice 6 weeks after the sham operation or MCAO, as described in previous studies [9,18].

### 4.3. Behavioral Tests

Neurological function was evaluated using CB-17/Icr-scid/scid Jcl mice that were randomly divided into four categories (mice injected with PBS after sham operation [sham/PBS group, *n* = 12], mice injected with PBS after MCAO [MCAO/PBS group, *n* = 12], mice administered h-iSCs after MCAO [MCAO/h-iSC group, *n* = 12], and mice administered h-MSCs after MCAO [MCAO/h-MSC group, *n* = 12]) 2–4 weeks after treatment (8–10 weeks post-MCAO or sham operation). The experiment and analysis were conducted in a blinded manner. Three weeks after finishing all behavioral tests, brains were resected as described below. In mice after MCAO, inappropriate samples (samples that did not have ischemic areas) were excluded, and statistical analysis was performed using appropriate samples (sham/PBS group, *n* = 12; MCAO/PBS group, *n* = 8; MCAO/h-iSC group, *n* = 12; MCAO/h-MSC group, *n* = 10).

Behavioral tests, including the basket, open-field, hot plate, and open-space swim tests, were performed, as described in previous studies [9,18]. In brief, the basket test was performed to evaluate sensorimotor deficits and motor coordination. A rectangular-shaped basket (30 × 30 × 40 cm) manufactured with the wire mesh plates with metal wires (diameter: 0.8 mm) woven in a grid pattern was used. Each mouse was placed in the center of the bottom of the basket and was allowed to explore freely for 10 s. The basket was then gently inverted and placed on a home cage with clean bedding. The latency to reach the floor of the home cage by climbing down the vertical mesh wall was measured. Each mouse was allowed to have three trials with an intertrial interval of 10 min.

The open-field test was performed to measure spontaneous locomotor activity. A transparent cubic box (30 × 30 × 30 cm) enclosed with white acrylic walls (width: 45 cm, height: 45 cm) was used as the open field. The mice were subjected individually to 10 min test sessions, and their behavior in the open-field arena was recorded with a digital video camera placed above the apparatus. A computerized video-based tracking system (Be-Chase ver. 2021; ISONIX Co., Ltd., Kobe, Japan) was used to measure the total distance traveled on the open-field arena.

The hot plate test was conducted to evaluate sensitivity to a painful stimulus. Each mouse was placed on a thermo-controllable aluminum plate (Model MK-350B, Muromachi Kikai Co., Tokyo, Japan) maintained at two different temperatures (56 °C and 60 °C ± 0.5 °C). To prevent tissue damage in mice, the latency to jump, with a cutoff value of 20 s, was recorded.

The open-space swimming test was conducted to evaluate depression-like status. We used a circular pool (inside diameter: 100 cm, depth: 45 cm) filled with water that was made opaque by adding nontoxic black paint to a depth of 30 cm. The water temperature was maintained at 27 °C ± 1 °C. The apparatus was illuminated using indirect lighting. The illumination level was 250 lux at the water surface. Each mouse was placed in the pool with its head facing the outer edge of the pool and was allowed to swim freely for 10 min. The behaviors of the mice were recorded with a digital video camera placed above the apparatus. Using the computerized video-based tracking system (Be-Chase ver. 2021; ISONIX Co., Ltd., Kobe, Japan), the total duration of immobility was calculated by summing up the time segments (seconds).

### 4.4. Immunohistochemistry

Immunohistochemistry was performed, as described in previous studies [6,9,18]. In brief, the mice were anesthetized intraperitoneally with a mixture of medetomidine, midazolam, and butorphanol. Then, they were transcardially perfused with 4% paraformaldehyde. The resected brain samples were further fixed with 4% paraformaldehyde, cryoprotected in 30% sucrose, and frozen at −80 °C. Then, they were cut into 20 μm coronal sections using a cryostat, and coronal brain sections (with a thickness of 20 μm) were stained with primary antibodies against GFP (1:2000, chicken, Abcam [ab13970], Cambridge, the UK) and mCherry (1:1000, rabbit, Abcam [ab167453]). Immunolabeling was visualized using Alexa Fluor 488- or 555-conjugated secondary antibodies (1:500, Molecular Probes, Eugene, OR, USA). Next, the nuclei were stained with 4′,6-diamidino-2-phenylindole (DAPI; 1:500, Kirkegaard & Perry Laboratories, Inc., Gaithersburg, MD, USA). Images were captured using a laser microscope (LSM780; Carl Zeiss AG, Oberkochen, Germany).

h-iSCs and h-MSCs were subjected to immunohistochemistry with an antibody against nestin (1:100, Santa Cruz, Biotechnology, Dallas, TX, USA), followed by Alexa Fluor 488-conjugated secondary antibodies (1:500, Molecular Probes, Eugene, OR, USA).

### 4.5. Cell Culture

Regionally derived endogenous NSPCs were extracted from the ischemic areas of the cortex of post-stroke mice. Then, they were maintained in DMEM/F12, including bFGF (20 ng/mL), EGF (20 ng/mL), 1% N2, and 2% FBS, as described in previous studies [9,18].

To investigate the effects of h-iSCs or h-MSCs on NSPCs, h-iSCs (GFP^+^ h-iSCs) or h-MSCs transfected with a GFP-expressing lentivirus (GFP^+^ h-MSCs) were cocultured with NSPCs transfected with NSPCs, as described in previous studies [9,18]. In brief, GFP^+^ h-iSCs (2.0 × 10^5^ cells/well) or GFP^+^ h-MSCs (2.0 × 10^5^ cells/well) were plated on 6-well dishes in DMEM/F12 medium containing bFGF, EGF, N2, and 2% FBS. After 1 day, NSPCs (1.0 × 10^4^ cells/well) were plated onto the dishes. On day 5 after coincubation, the samples were fixed and subjected to immunohistochemistry. NSPCs (1.0 × 10^4^ cells/well), which were used as controls, were monocultured in the same dishes for 5 days. Next, immunohistochemistry was performed using antibodies against GFP (1:2000, chicken, Abcam) and nestin (1:200, mouse, Millipore, St. Louis, MO, USA). Then, the samples were incubated with Alexa Fluor 488- or 555-conjugated secondary antibodies (1:500, Molecular Probes), as described in previous studies [9,18]. Then, the number of nestin^+^ NSPCs (GFP^−^/nestin^+^ cells) was measured in monocultured NSPCs (controls), NSPCs cocultured with GFP^+^ h-iSCs, or GFP^+^ h-MSCs using 12 data points (four areas/sample, three samples/group [*n* = 3]).

To investigate the effects of h-iSCs or h-MSCs on NSPCs, GFP^+^ h-iSCs or GFP^+^ h-MSCs were cocultured with NSPCs transfected with an mCherry-expressing lentivirus (mCherry^+^ NSPCs), as mentioned in the previous text [9,18]. Briefly, GFP^+^ h-iSCs (2.0 × 10^5^ cells/well) or GFP^+^ h-MSCs (2.0 × 10^5^ cells/well) were plated on 6-well dishes in DMEM/F12 medium containing bFGF, EGF, N2, and 2% FBS. After 1 day, mCherry^+^ NSPCs (1.0 × 10^4^ cells/well) were plated onto the same dishes. On day 5 after coincubation, mCherry^+^ NSPCs were selectively collected via FACS and subjected to microarray analysis.

Next, to investigate the effects of NSPCs on h-iSCs, GFP^+^ h-iSCs were cocultured with mCherry^+^ NSPCs, as described in previous studies [9,18]. In brief, GFP^+^ h-iSCs (2.0 × 10^5^ cells/well) were plated on 6-well dishes in DMEM/F12 medium containing bFGF, EGF, N2, and 2% FBS. After 1 day, GFP^+^ h-iSCs were cultured alone or cocultured with mCherry^+^ NSPCs (1.0 × 10^4^ cells/well). On day 6 after incubation, GFP^+^ h-iSCs were selectively collected via FACS and subjected to microarray analysis.

Similarly, to investigate the effects of NSPCs on h-MSCs, GFP^+^ h-MSCs were cocultured with mCherry^+^ NSPCs, as described in previous studies [9,18]. In brief, GFP^+^ h-MSCs (2.0 × 10^5^ cells/well) were plated on 6-well dishes in DMEM/F12 medium containing bFGF, EGF, N2, and 2% FBS. After 1 day, GFP^+^ h-MSCs were cultured alone (control) or cocultured with mCherry^+^ NSPCs (1.0 × 10^4^ cells/well). On day 6 after incubation, GFP^+^ h-MSCs were selectively collected via FACS and subjected to microarray analysis.

To investigate the neural network formation between h-iSCs and NSPCs or that between h-MSCs and NSPCs, GFP^+^ h-iSCs (2.0 × 10^4^ cells/well) or GFP^+^ h-MSCs (2.0 × 10^4^ cells/well) were plated on poly-L-lysine-coated 24-well dishes in neurobasal medium (Thermo Fisher Scientific) containing bFGF, B-27 supplement (Thermo Fisher Scientific), and 2% FBS. On the following day, NSPC-derived neurospheres (approximately 5–10 spheres/well) were seeded on each dish. On day 14 after seeding, the samples were fixed and immunostained with antibodies against GFP (1:2000, chicken, Abcam) and MAP2 (1:1000, rabbit, Millipore). Immunolabeling was visualized using Alexa Fluor 488- or 555-conjugated secondary antibodies (1:500, Molecular Probes). Then, the nuclei were counterstained with 4′,6-diamidino-2-phenylindole (DAPI; 1:500, Kirkegaard & Perry Laboratories, Inc.). Images were captured using a laser scanning microscope (LSM780; Carl Zeiss AG).

Then, the ratios of NSPC-derived differentiated neurons (GFP^−^/MAP2^+^ cells to GFP^−^/DAPI^+^ cells) and h-iSC-derived neurons (GFP^+^/MAP2^+^ cells to GFP^+^/DAPI^+^ cells) were analyzed using nine data points (three areas/sample, three samples [*n* = 3]), as described in previous studies [9,18]. Similarly, the ratios of NSPC-derived differentiated neurons (GFP^−^/MAP2^+^ cells to GFP^−^/DAPI^+^ cells) and h-MSC-derived neurons (GFP^+^/MAP2^+^ cells to GFP^+^/DAPI^+^ cells) were analyzed using nine data points (three areas/sample, three samples [*n* = 3]). The numbers of interactions between NSPC-derived neurons (GFP^−^/MAP2^+^ cells) and h-iSC-derived neurons (GFP^+^/MAP2^+^ cells) or those between NSPC-derived neurons (GFP^−^/MAP2^+^ cells) and h-MSC-derived neurons (GFP^+^/MAP2^+^ cells) were analyzed using nine data points (three areas/sample, three samples [*n* = 3]).

### 4.6. Microarray Analysis

Total RNA was isolated from NSPCs (cocultured with h-iSCs or h-MSCs), h-iSCs (h-iSCs alone or h-iSCs cocultured with NSPCs), and h-MSCs (h-MSCs alone or h-MSCs cocultured with NSPCs) using the RNeasy Micro Kit (Qiagen, Hilden, Germany), as described in previous studies [6,9,18]. Then, RNA samples (*n* = 1, for each group) were subjected to microarray analysis, and the results were analyzed using the affymetrix transcriptome analysis console, as described in previous studies [6,9,18]. Pathway analysis was performed using WikiPathways, as described in previous reports [18,46]. GO analysis was performed using Metascape GO tool [47].

### 4.7. Single-Cell RNA Sequencing Analysis

h-iSCs and h-MSCs were subjected to scRNA-seq using the ICELL8 System by a contract service (Takara Bio Inc., Shiga, Japan), as described in previous studies [9,48].

### 4.8. Statistical Analysis

Data were presented as means ± standard errors of the mean. Between-group differences were evaluated using the Student’s *t*-test. Comparisons among three or more groups were performed using one-way analysis of variance, followed by the post-hoc tests, as described in previous studies [9,18]. A *p* value of <0.05 was considered statistically significant.

## 5. Conclusions

A comparative preclinical study using h-iSCs and h-MSCs showed that both h-iSC transplantation and h-MSC transplantation improved the neurological functions of mice after ischemic stroke. However, compared with h-MSC transplantation, h-iSC transplantation was associated with a greater neurological improvement. Although further studies must be performed to evaluate the actual mechanism, the current study showed that h-iSC transplantation can be a novel therapy for treating patients with stroke.

## Data Availability

The data supporting this article will be shared by the corresponding author upon reasonable request.

## Supplementary Materials

The following supporting information can be downloaded at: https://www.mdpi.com/article/10.3390/ijms252212065/s1.
