# Supplementary material for: Human-Brain-Derived Ischemia-Induced Stem Cell Transplantation Is Associated with a Greater Neurological Functional Improvement Compared with Human-Bone Marrow-Derived Mesenchymal Stem Cell Transplantation in Mice After Stroke
Source: Int J Mol Sci. 2024 Nov 10;25(22):12065. doi: 10.3390/ijms252212065 (PMC11593343; doi:10.3390/ijms252212065)
Supplement: Supplementary file 1 [file ijms-25-12065-s001.zip › ijms-3241701-supplementary.pdf]

Supplementary Figure S1

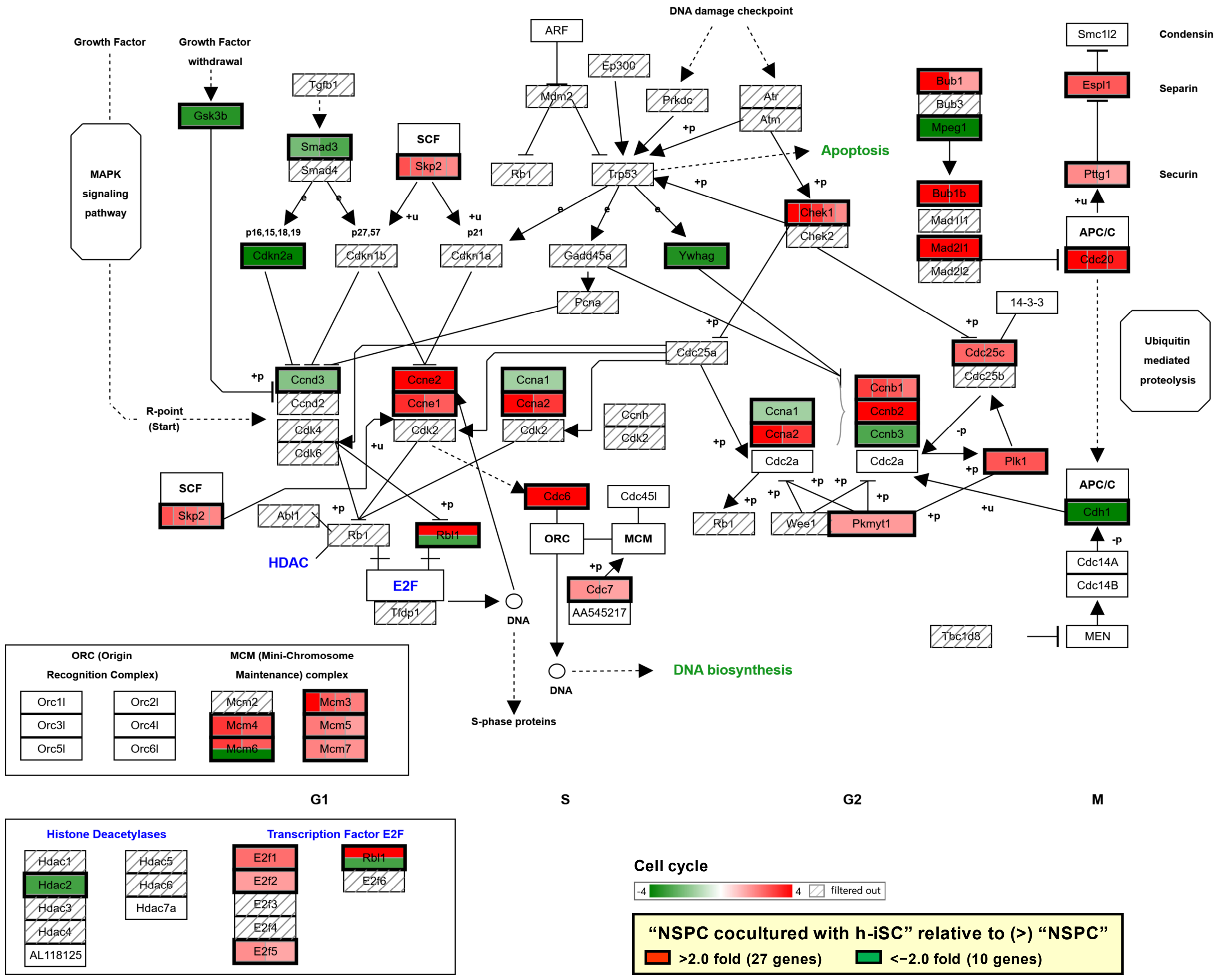

Supplementary Figure S2

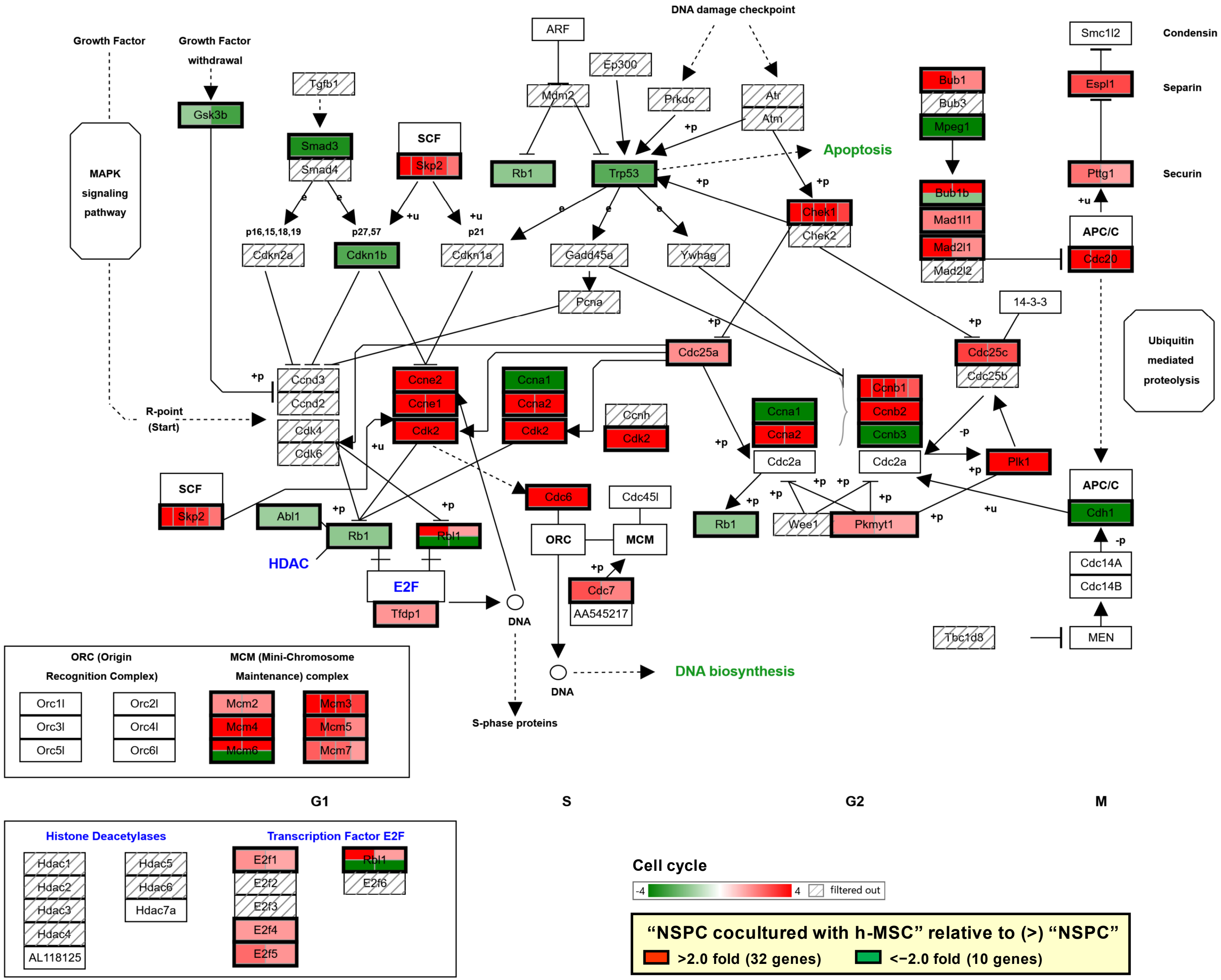

Supplementary Figure S3

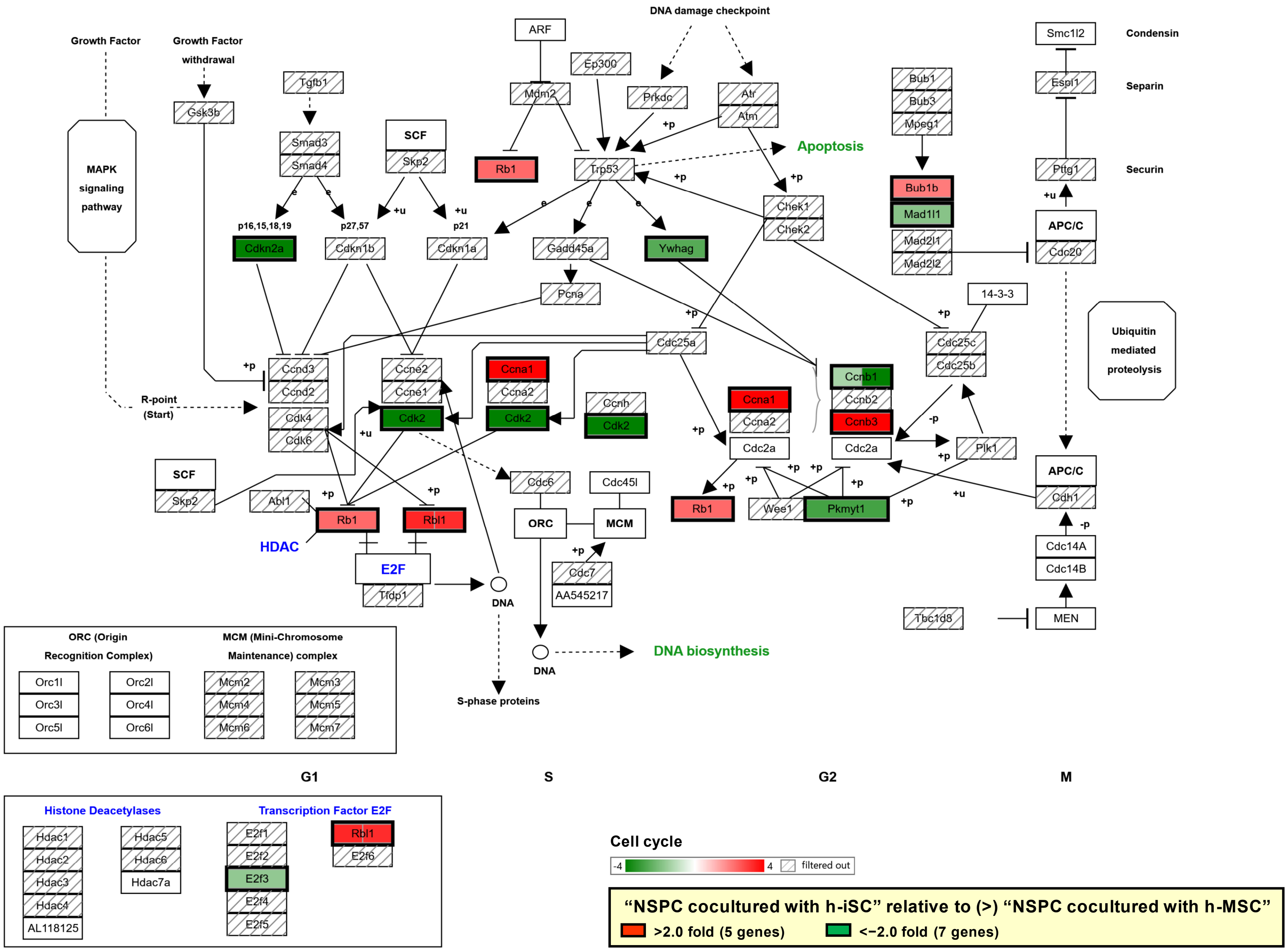

Supplementary Figure S4

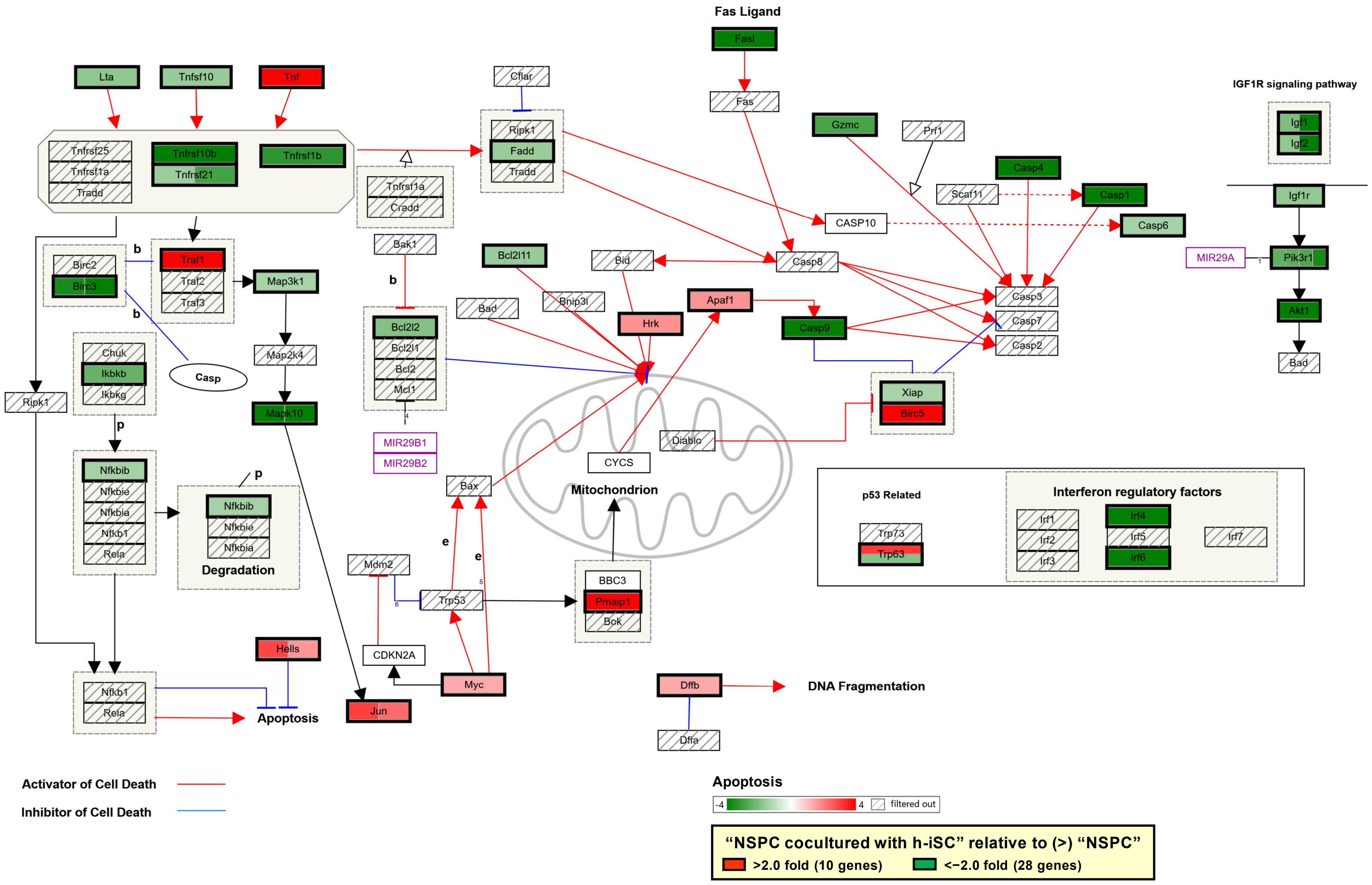

Supplementary Figure S5

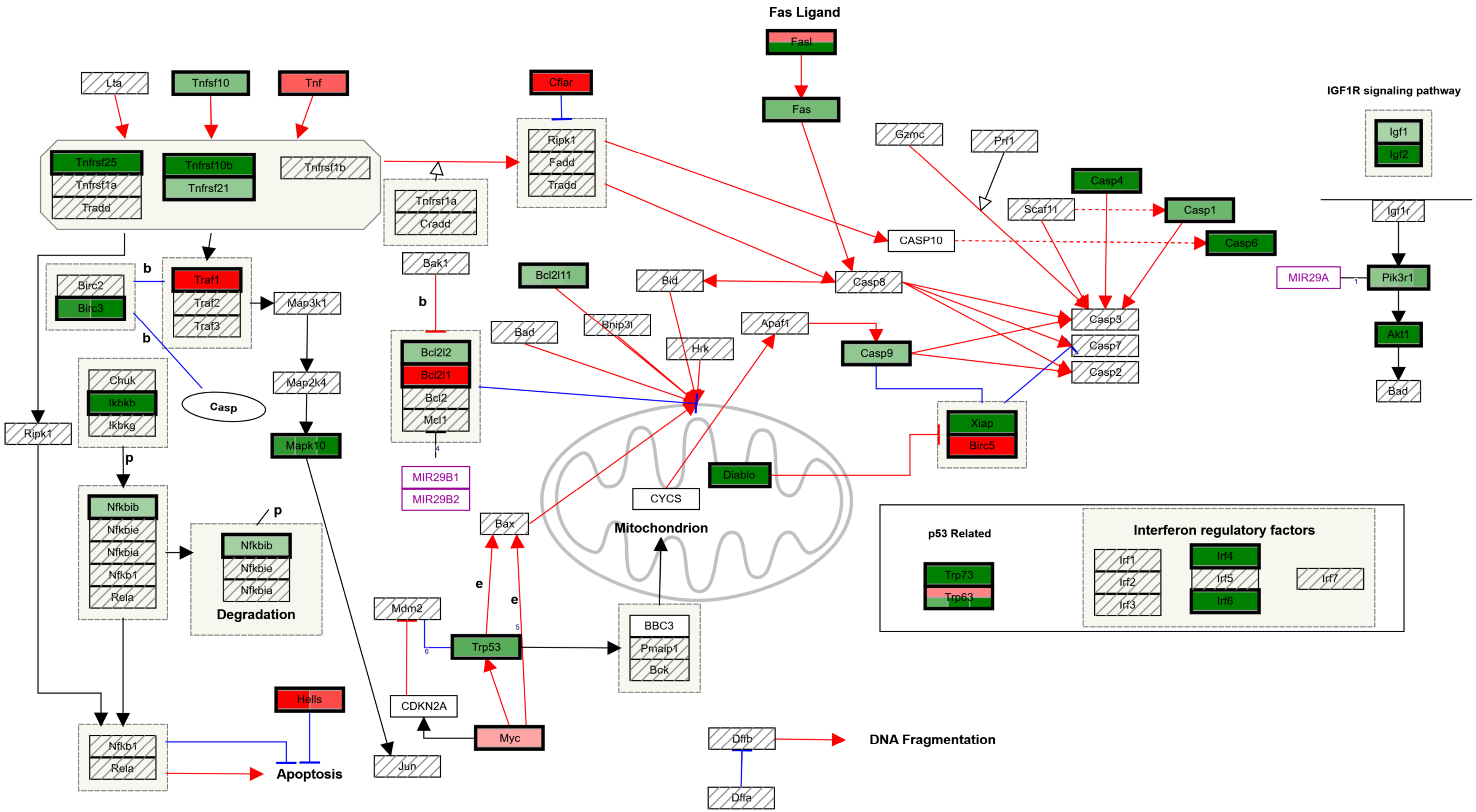

Activator of Cell Death

Inhibitor of Cell Death

Apoptosis

-4 4 filtered out

"NSPC cocultured with h-MSC" relative to (>) "NSPC"

>2.0 fold (8 genes) <−2.0 fold (26 genes)

Supplementary Figure S6

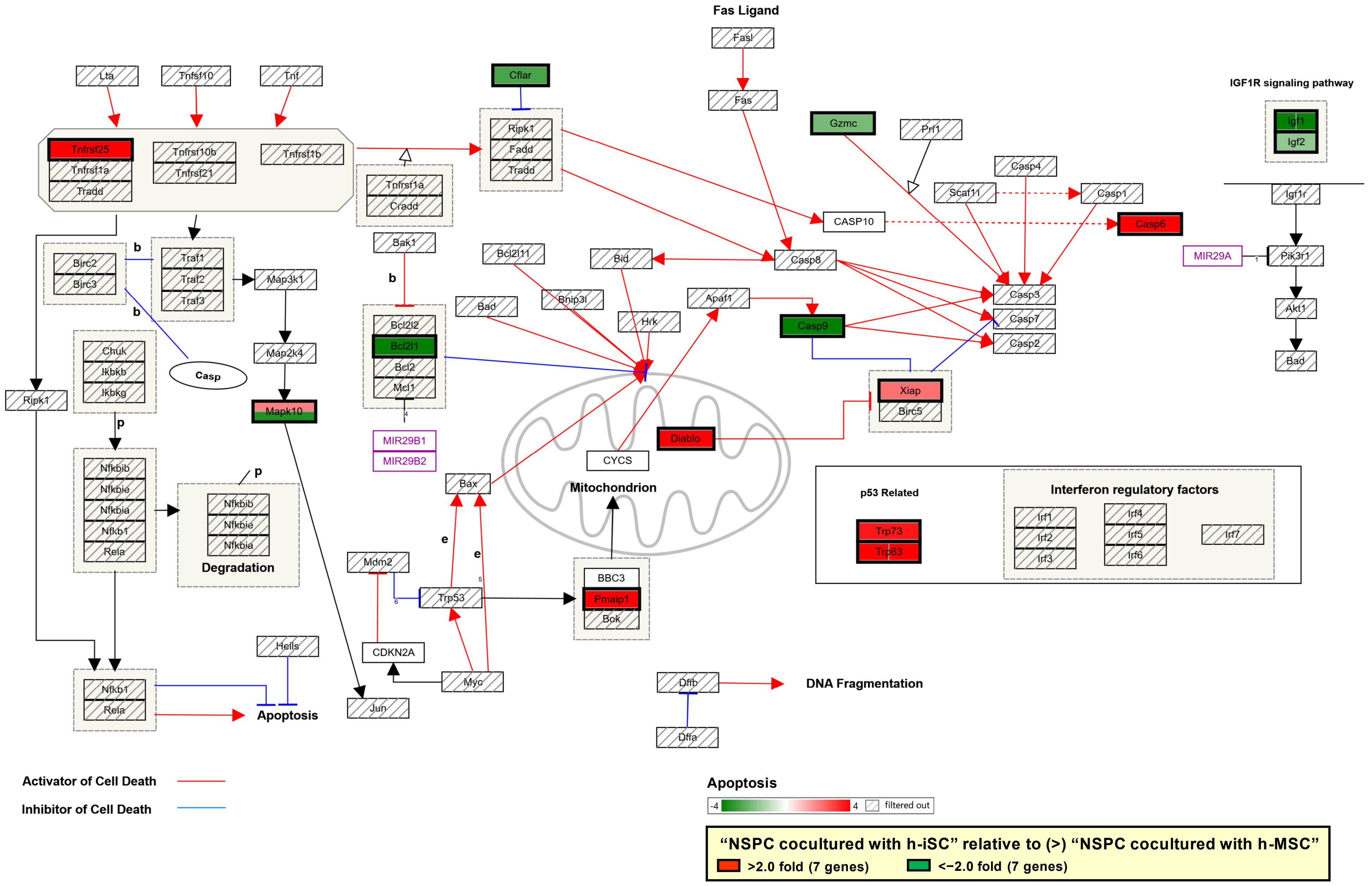

Supplementary Figure legends

Figure 1

Pathway analysis of cell cycle-related genes in NSPCs cocultured with h-iSCs relative to NSPCs monocultures.

Figure 2

Pathway analysis of cell cycle-related genes in NSPCs cocultured with h-MSCs relative to NSPCs monocultures.

Figure 3

Pathway analysis of cell cycle-related genes in NSPCs cocultured with h-iSCs relative to NSPCs cocultured with h-MSCs.

Figure 4

Pathway analysis of apoptosis-related genes in NSPCs cocultured with h-iSCs relative to NSPCs monocultures.

Figure 5

Pathway analysis of apoptosis-related genes in NSPCs cocultured with h-MSCs relative to NSPCs monocultures.

Figure 6

Pathway analysis of apoptosis-related genes in NSPCs cocultured with h-iSCs relative to NSPCs cocultured with h-MSCs.
